# CRP-cAMP mediates silencing of *Salmonella* virulence at the post-transcriptional level

**DOI:** 10.1371/journal.pgen.1007401

**Published:** 2018-06-07

**Authors:** Youssef El Mouali, Tania Gaviria-Cantin, María Antonia Sánchez-Romero, Marta Gibert, Alexander J. Westermann, Jörg Vogel, Carlos Balsalobre

**Affiliations:** 1 Department of Genetics, Microbiology and Statistics, School of Biology, Universitat de Barcelona, Barcelona, Spain; 2 Department of Genetics, Universidad de Sevilla, Sevilla, Spain; 3 RNA Biology Group, Institute for Molecular Infection Biology (IMIB), University of Würzburg, Würzburg, Germany; 4 Helmholtz Institute for RNA-based Infection Research (HIRI), Würzburg, Germany; Swiss Federal Institute of Technology Lausanne (EPFL), SWITZERLAND

## Abstract

Invasion of epithelial cells by *Salmonella enterica* requires expression of genes located in the pathogenicity island I (SPI-1). The expression of SPI-1 genes is very tightly regulated and activated only under specific conditions. Most studies have focused on the regulatory pathways that induce SPI-1 expression. Here, we describe a new regulatory circuit involving CRP-cAMP, a widely established metabolic regulator, in silencing of SPI-1 genes under non-permissive conditions. In CRP-cAMP-deficient strains we detected a strong upregulation of SPI-1 genes in the mid-logarithmic growth phase. Genetic analyses revealed that CRP-cAMP modulates the level of HilD, the master regulator of *Salmonella* invasion. This regulation occurs at the post-transcriptional level and requires the presence of a newly identified regulatory motif within the *hilD* 3’UTR. We further demonstrate that in *Salmonella* the Hfq-dependent sRNA Spot 42 is under the transcriptional repression of CRP-cAMP and, when this transcriptional repression is relieved, Spot 42 exerts a positive effect on *hilD* expression. *In vivo* and *in vitro* assays indicate that Spot 42 targets, through its unstructured region III, the 3’UTR of the *hilD* transcript. Together, our results highlight the biological relevance of the *hilD* 3’UTR as a hub for post-transcriptional control of *Salmonella* invasion gene expression.

## Author summary

*Salmonella* infection is one of the major causes of foodborne illness worldwide. During infection, *Salmonella* expresses a set of virulence genes encoded in discrete regions of the genome. The expression of these genes is tightly regulated, being specific for different stages of the *Salmonella* infection process. While many regulatory mechanisms that lead to the activation of infection-related gene expression have been described, little is known about silencing mechanisms under conditions when expression is not needed and may rather represent a burden than a benefit for the bacterial fitness. Here, we report a condition-specific silencing mechanism of bacterial virulence. That is, the global transcriptional regulator CRP-cAMP represses, indirectly through a post-transcriptional mechanism, the expression of the major *Salmonella* virulence regulator HilD. In bacteria, post-transcriptional regulation has so far been mainly focused on 5’ untranslated regions (5’UTR). Remarkably, here we describe a molecular mechanism targeting the 3’untranslated region (3’UTR) of the mRNA of the major regulator of *Salmonella* virulence by a small non-coding RNA under the transcriptional control of the global regulator CRP-cAMP. Our data highlight the importance of 3’UTR in the regulation of gene expression in bacteria.

## Introduction

*Salmonella enterica* serovar Typhimurium is a prevalent gastrointestinal pathogen. Upon arrival in the intestinal lumen, *Salmonella* is able to both invade epithelial cells and survive within phagocytic cells. Genomic studies revealed the presence of several pathogenicity islands in the *Salmonella* chromosome (SPIs). Among them, SPI-1 and SPI-2 are the best studied and known to encode factors required for invasion of non-phagocytic cells and survival within macrophages, respectively [1,2]. Induction of virulence programs is generally associated with significant energetic costs for the bacterial cell. For example, induction of SPI-1 under non-infectious conditions *in vitro* has a negative impact on cell physiology, resulting in a deleterious effect on *Salmonella*’s growth [3]. Consequently, the expression of virulence programs is generally tightly regulated and induction occurs only upon sensing of a variety of defined environmental and physiological signals.

The complex regulatory circuit that controls SPI-1 expression has attracted much attention [2,4] and become a model to understand how the activities of multiple molecular factors converge to achieve a precise timing of virulence gene activation. The majority of the multiple signal transduction systems that modulate SPI-1 regulation converge at the level of HilA expression, a SPI-1 encoded transcriptional regulator required for the expression of most SPI-1 genes [5]. *Salmonella* does not express HilA when it is growing exponentially in LB cultures, a condition stated as non-permissive in the present study. However, HilA expression is induced at early stationary phase, when growth conditions become nutrient-limiting [6], a condition here referred to as SPI-1-permissive. Transcription of *hilA* is controlled by three AraC-like transcriptional activators: HilD, HilC and RtsA. The first two are encoded within SPI-1 itself, while RtsA is encoded outside this locus [7]. HilD, HilC and RtsA form a feed-forward regulatory loop, whereby each activator induces the two other genes, but also auto-regulates its own expression [8]. This regulatory triad responds to a wide range of physiological and environmental stimuli that are sensed by a variety of cellular factors, including both global and specific regulators (as reviewed by Fabrega and Vila [2]). Within this triad, a prominent role has been attributed to HilD, the main target for signaling pathways controlling SPI-1 expression [8,9]. Regulatory mechanisms have been described, acting at all levels of *hilD* gene expression—transcriptional, post-transcriptional, translational and post-translational [10–13]. Most studies focused on the mechanisms required for full induction of SPI-1 genes, whereas very little is known on the regulatory pathways involved in the shutdown of the SPI-1 expression under non-permissive conditions. Here, we report that general transcription factor CRP is required to silence SPI-1 genes in exponential growing cells and propose a new regulatory axis formed by CRP and the broadly conserved small RNA (sRNA) Spot 42 that contributes to growth phase-specific activation of SPI-1 genes.

CRP is a global transcriptional regulator that acts as a metabolic sensor upon binding of intracellular cAMP (cyclic adenosine monophosphate), which is synthesized by the adenylate cyclase CyaA [14]. CRP-cAMP-deficient *Salmonella* strains are unable to secrete SPI-1 T3SS effector proteins and are avirulent in a mouse model, suggesting a role for CRP-cAMP in the regulation of *Salmonella* virulence [15,16]. Indeed, CRP-cAMP indirectly regulates virulence by affecting the post-transcriptional regulation of *hilD*. The sRNAs CsrB and CsrC are under the transcriptional control of Bar/SirA and are upregulated in a *crp* knockout mutant. CsrB and CsrC are antagonists of CsrA, a post-transcriptional repressor of *hilD* mRNA [10,17,18]. Therefore, in early stationary phase (permissive conditions for SPI-1 expression), CRP-cAMP generally promotes SPI-1 expression by indirectly repressing the activity of CsrA [17–19]. Here, we report that in mid-logarithmic growth phase (non-permissive conditions), CRP-cAMP represses *hilD* expression by a mechanism requiring Hfq and the 3’UTR of *hilD* mRNA. Given the established primary role of Hfq in mediating the base pairing interactions of sRNAs [20,21], it is tempting to speculate that *hilD* may be post-transcriptionally regulated by a CRP-cAMP controlled sRNA; this control, however, would be unusual in light of the fact that almost all Hfq-associated sRNAs characterized to date recognize mRNAs in the 5’ region. Of several candidates for CRP-cAMP-dependent sRNAs known in enteric bacteria [22], Spot 42 has been best characterized in *Escherichia coli*, where together with CRP-cAMP, it forms a multi-output feedforward loop to enact catabolite repression [23–27]. In *Salmonella*, Spot 42 has been known as one of the most abundant Hfq-associated sRNAs in fast-growing cells [28], but except for a repression of the sugar-related *mglB* mRNA [29] its activity has not been characterized.

By dissecting the molecular mechanism of CRP-cAMP-mediated SPI-1 repression in exponentially growing *Salmonella*, we here reveal novel mechanisms by which sRNAs target mRNAs. Our data point towards an unusual post-transcriptional stimulation of the *hilD* mRNA by Spot 42. Different from other *trans*-acting sRNA characterized, Spot 42-mediated activation occurs in the 3’ UTR of the *hilD* mRNA, adding to a growing appreciation of mRNA 3’ ends as sites for post-transcriptional control in bacteria.

## Results

### CRP-cAMP represses SPI-1 expression

**Fig 1:**
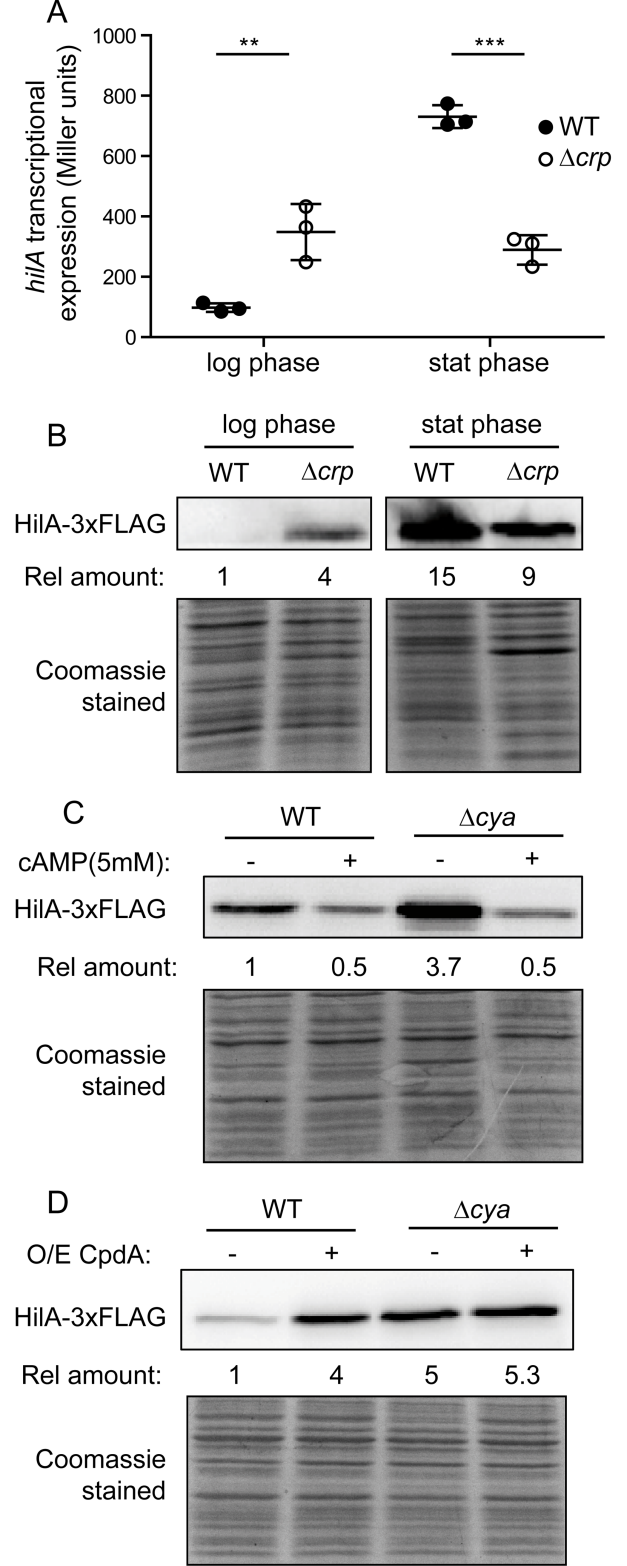
CRP-cAMP represses *hilA* expression in mid-logarithmic growing cells. (A) Transcriptional expression of *hilA* in a wild-type (WT) and a Δ*crp* derivative strain. β-galactosidase activity from a *hilA*-*lacZ* fusion was assessed in LB cultures grown at 37°C up to either mid-logarithmic (OD_600nm_ 0.4) or early stationary (OD_600nm_ 2.0) phase of growth. Data from three independent experiments are averaged and the standard deviation is shown. **, *p*< 0.01; ***, *p*< 0.001. (B) Immuno-detection of HilA-3xFLAG protein was performed on whole cell extracts of the WT and Δ*crp* derivative strains, grown as in A. (C). Immuno-detection of HilA-3xFLAG on whole cell extracts from cultures of the WT and Δ*cya* derivative strains in the absence (-) or presence (+) of cAMP (5 mM). Cultures were grown in LB at 37°C up to an OD_600nm_ of 0.4. (D) Effect of over-expressing the cAMP-phosphodiesterase CpdA. Immunodetection of HilA-3xFLAG was performed on cultures of the WT and a Δ*cya* derivative strain carrying either pTrc99a (-, control vector) or pCpdA (+, pTrc99a+*cpdA*). Cultures were grown as in C in LB supplemented with IPTG (0.1 mM). In B, C and D the relative amount of HilA-3xFLAG is indicated. In each case the reference value was set as one. Coomassie Blue staining of the whole cell extracts serve as loading controls. Full length images of the Western blots, including molecular mass markers, are shown in S12 Fig.

To characterize the role of the metabolic sensor CRP-cAMP in SPI-1 expression, we monitored transcription of the main regulator HilA in wild-type and Δ*crp* derivative strains grown in LB at 37°C. The expression pattern was determined in mid-logarithmic cultures (OD_600nm_ 0.4, non-permissive conditions for SPI-1 expression) and at early stationary phase (OD_600nm_ 2.0, permissive conditions for SPI-1 expression) (Fig 1A). Consistent with previous reports, a growth phase dependent profile in SPI-1 expression was observed [6]. In the wild-type strain, *hilA* expression levels were 8-fold higher at early stationary phase when compared to mid-logarithmic cultures. Remarkably, we also observed a growth-phase dependent effect of the Δ*crp* mutation. In agreement with previous work [18], the Δ*crp* mutation reduces *hilA* transcription in early stationary phase. In mid-logarithmic cultures, however, the Δ*crp* mutation caused an upregulation of *hilA* expression (4-fold, as compared to the wild-type). Using a chromosomally encoded FLAG-tagged HilA variant, these transcriptional profiles were corroborated on the protein level. More HilA protein accumulated in the Δ*crp* mutant in mid-logarithmic cultures and less in early stationary cultures, relative to wild-type levels (Fig 1B).

CRP is active upon binding of the cofactor cAMP [14]. Therefore, lack of either CRP or cAMP should have a similar effect on SPI-1 expression. HilA levels were monitored in a Δ*cya* mutant strain, which is deficient in the synthesis of cAMP (Fig 1C). As expected, Δ*cya* mutation caused an almost 4-fold increase in HilA levels in mid-logarithmic phase cells. Chemical complementation was performed by monitoring HilA abundance after addition of cAMP (Fig 1C). An 8-fold decrease in HilA levels was observed when cAMP was added to cultures of the Δ*cya* strain. Interestingly, when cAMP was added to a culture of a *cya*^+^ (i.e. wild-type) strain, a 2-fold drop in HilA levels was observed. These results may indicate that the intracellular cAMP levels were not saturating all CRP molecules. Consequently, external addition of the cofactor to wild-type cultures would lead to an increase in the number of CRP-cAMP complexes, causing further repression of HilA expression.

To further corroborate the involvement of cAMP in the control of HilA expression, the intracellular levels of cAMP were lowered by ectopically over-expressing CpdA in *Salmonella*, a putative cAMP phosphodiesterase [30]. Over-expression of CpdA, confirmed by immunodetection (S1 Fig), caused a 4-fold increase in HilA expression in the wild-type background. This clearly depended on cAMP turnover, since CpdA over-expression had no effect in a Δ*cya* strain (Fig 1D).

### Impact on SPI-1-encoded effector proteins

**Fig 2:**
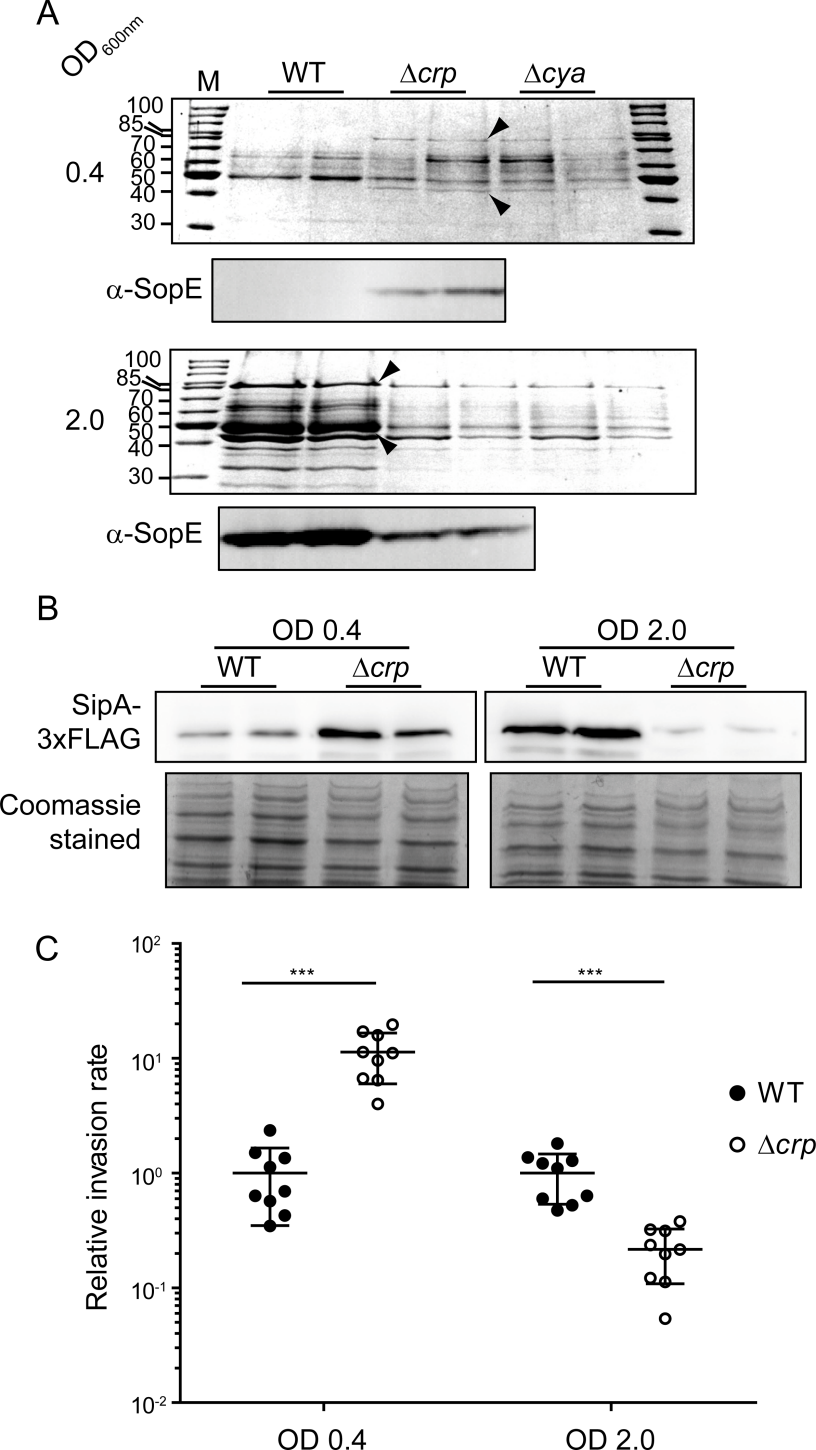
Effect of *crp* and *cya* deletion on the expression of SPI-1 effector proteins. (A) Proteins from cell-free supernatants from cultures of the wild-type (WT), Δ*crp* and Δ*cya* derivative strains grown up to mid-logarithmic (OD_600nm_ 0.4) or early stationary (OD_600nm_ 2.0) phase were TCA precipitated. The resulting extracts were analyzed by SDS-PAGE and Coomassie Blue staining. Size in kDa of the molecular mass marker (M) is indicated. Arrowheads indicate the protein bands corresponding to SipA (upper band) and SipC (lower band). Immunodetection of SopE protein (α-SopE) was performed in WT and Δ*crp* extracts. (B) Immunodetection of SipA-3xFLAG was performed on whole cell extracts from cultures of the WT and Δ*crp* derivative strains grown as in A. Coomassie Blue stainings of the whole cell extracts serve as loading controls. In A and B, extracts from two independent cultures of the same strain were analyzed. Full length images of the Western blots, including molecular mass markers, are shown in S12 Fig. (C) Invasion of HeLa cells. Cultures of the WT and Δ*crp* strains were grown as in A and assessed for invasion of HeLa cells. Invasion rates were calculated and given as relative rate, the reference value of WT was set to one in both mid-logarithmic and early stationary phase cultures. The invasion rates were 0.7 x 10^−3^ and 2.4 x 10^−2^ for the WT in mid-logarithmic and early stationary phase, respectively. ***, *p*< 0.001.

HilA regulates the transcriptional expression of most SPI-1 genes, including those required for the synthesis of a type III secretion system (T3SS) and several effector proteins that are translocated to the host cell during *Salmonella* infection [2]. A Δ*hilA* mutation impairs secretion of SPI-1 effector proteins [31]. Comparative studies of the secreted protein profile between wild-type and Δ*hilA* strains were performed to identify protein bands corresponding to SPI-1 effectors (S2 Fig). Major protein bands exclusively detected in extracts of the wild-type strain were identified by LC-MS/MS as the SPI-1-encoded proteins SipA and SipC. The secretome of wild-type, Δ*crp* and Δ*cya* derivative strains was characterized in LB cultures grown to mid-logarithmic and early stationary phase (Fig 2A). Consistent with previous reports [16], CRP-cAMP-deficient cells in early stationary phase showed a lower amount of those secreted proteins. Yet, CRP-cAMP-deficient *Salmonella* hyper-secreted SPI-1 effector proteins in mid-logarithmic phase cultures. The Δ*crp*-dependent overproduction in mid-logarithmic phase of the larger protein, the effector protein SipA, was confirmed by using a SipA-3xFLAG variant (Fig 2B).

HilA has also been reported to regulate the expression of SopE, an effector protein that is encoded outside the SPI-1 locus but it is secreted by the SPI-1 encoded T3SS [32]. SopE levels were monitored in secreted protein extracts of wild-type and Δ*crp* mutant strains (Fig 2A). Again, the Δ*crp* strain secreted more SopE protein in the mid-logarithmic phase and less in early stationary phase, as compared to wild-type. The fact that CRP-cAMP-mediated repression of *hilA* expression has a concomitant effect on the expression and secretion of SPI-1 effector proteins highlights the biological relevance of CRP-cAMP in the control of *Salmonella* virulence under non-permissive conditions. In support of this notion, a Δ*crp* mutant grown to mid-logarithmic phase prior to infection, invaded HeLa cells more efficiently (>10-fold) than the wild-type (Fig 2C). In contrast, the wild-type strain showed a higher rate (>4.5-fold) than the Δ*crp* derivative when cultures were grown to early stationary phase prior to infection.

### CRP-cAMP regulation of SPI-1 occurs upstream of HilA by repression of *hilD*, *hilC* and *rtsA*

**Fig 3:**
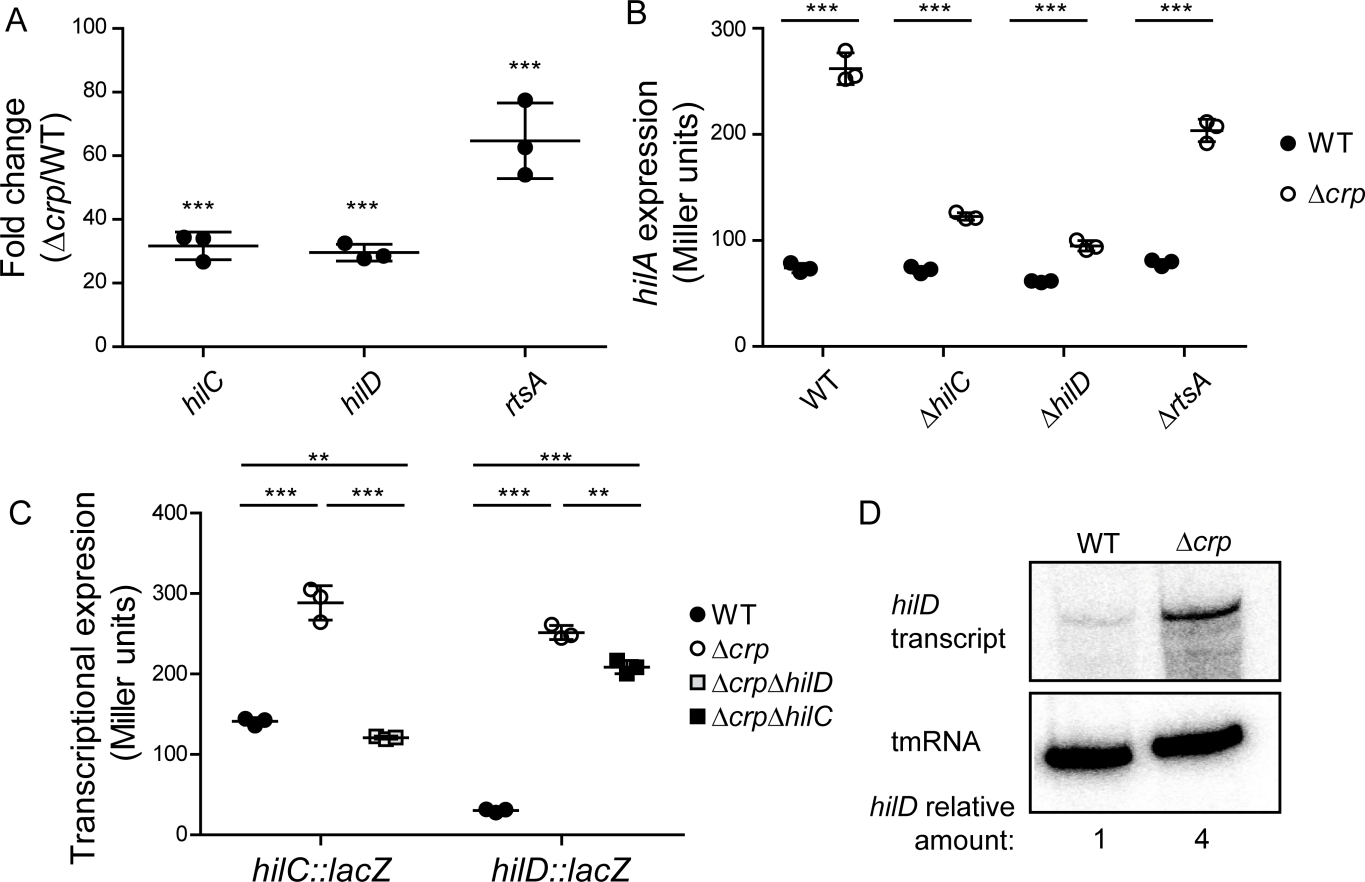
Effect of *crp* deletion on the expression of upstream activators of *hilA*. (A) Relative quantification of *hilD*, *hilC* and *rtsA* mRNA by qRT-PCR. Total RNA samples were extracted from cultures of the wild-type (WT) and Δ*crp* derivative strains. Results are expressed as fold changes between WT and Δ*crp*. Detection of *gapA* (GAPDH) was used as an internal control (see Material and Methods). (B) *hilA* transcriptional expression (β-galactosidase activity) was monitored in *crp*^+^ and Δ*crp* strains in different genetic backgrounds: WT, Δ*hilC*, Δ*hilD* and Δ*rtsA*. (C) *hilC* and *hilD* transcriptional expression (β-galactosidase activity) was monitored. For *hilC*, WT, Δ*crp* and Δ*crp* Δ*hilD* derivative strains carrying a *hilC*-*lacZ*, chromosomal fusion were used. For *hilD*, WT, Δ*crp* and Δ*crp* Δ*hilC* carrying the *hilD*1235-*lacZ* (*hilD*^+^, 3’UTR^+^) were used. In A, B and C, data correspond to the average and standard deviation of three independent experiments. **, *p*< 0.01; ***, *p*< 0.001. (D) Northern blot analysis for *hilD* mRNA. Total RNA samples were extracted from cultures of the WT and Δ*crp* strains. tmRNA was detected as loading control. For quantification, the ratio [*hilD* mRNA/tmRNA] was calculated. Full length image of the northern blot is shown in S12 Fig. In all cases cultures were grown in LB at 37°C up to an OD_600nm_ of 0.4.

Three AraC-like transcriptional activators, HilD, HilC and RtsA, are directly involved in *hilA* activation [8]. To determine at which level CRP-cAMP controls SPI-1 through HilA, the expression of *hilD*, *hilC* and *rtsA* mRNA was monitored. RNA was extracted from mid-logarithmic cultures (OD_600nm_ 0.4) of both wild-type and Δ*crp* derivative strains and the relative amounts of mRNA of all three AraC-like regulators were determined by qRT-PCR. As shown in Fig 3A, in the Δ*crp* mutant higher transcripts levels of *hilD*, *hilC* and *rtsA* were detected than in the wild-type, indicating that the effect of CRP-cAMP on SPI-1 expression occurs upstream of HilA. The *hilA* transcript was also monitored by qRT-PCR as a control; as expected, it too over-accumulated in the Δ*crp* strain (S3 Fig).

HilD, HilC and RtsA form a feed-forward regulatory loop to stimulate *hilA* expression. In order to elucidate the direct target of CRP-mediated regulation of SPI-1, mutants of each of the three regulators in a *crp* proficient and deficient background were generated and *hilA* expression monitored in mid-logarithmic cultures (Fig 3B). In the *crp*^+^ strains, *hilA* expression was very low in all genetic backgrounds, further validating the silenced *hilA* expression during logarithmic growth. The *hilA* derepression in the absence of CRP was altered in the different mutants. Remarkably, in the absence of HilC and HilD the derepression of *hilA* transcription was greatly reduced. The expression of *hilC* and *hilD* was further studied using transcriptional *lacZ* fusions. As expected, both *hilC* and *hilD* were deregulated in a Δ*crp* mutant background (Fig 3C). Particularly, HilD seems to be required for the observed upregulation of *hilC* in the Δ*crp* background, whereas *hilD* induction does not require HilC. Taken together, these results suggest that HilD is the direct target of CRP-cAMP-mediated regulation of SPI-1 expression. Northern blot detection further corroborates an increase in the levels of *hilD* mRNA in the Δ*crp* strain as compared to wild-type in mid-logarithmic phase (Fig 3D).

### CRP-cAMP-mediated transcriptional regulation of HilD requires the 3’UTR of the *hilD* transcript

**Fig 4:**
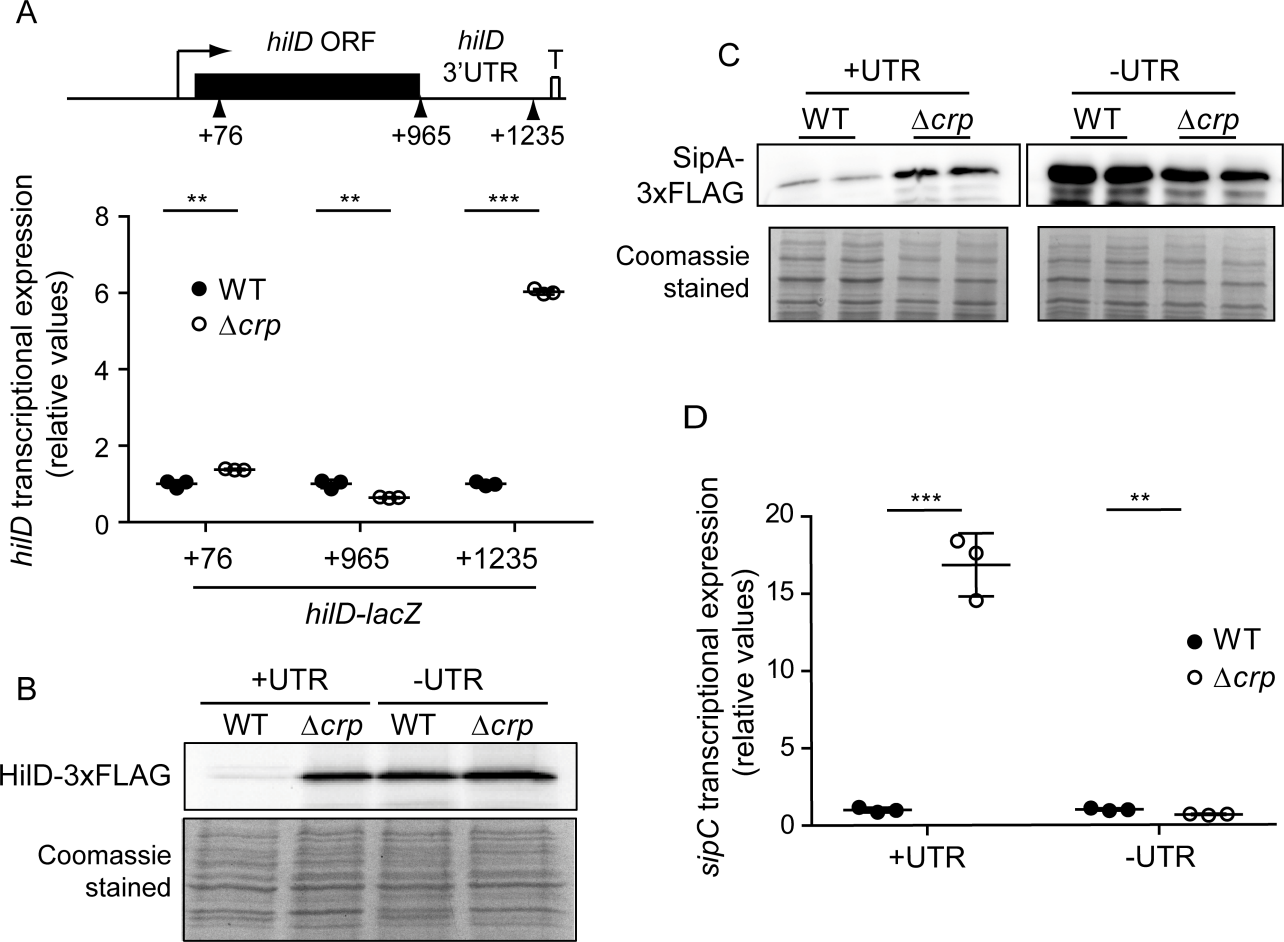
CRP-cAMP-mediated repression of HilD in mid-logarithmic growth requires the 3’UTR of *hilD*. (A) *hilD* transcriptional expression was monitored using three different *hilD-*lacZ reporter fusions; these comprise either *hilD* to position +76 (within the *hilD* ORF), to position +965 (including the full-length *hilD* ORF), or to position +1,235 (including the *hilD* ORF and the *hilD* 3’UTR). Transcriptional studies were performed in the wild-type (WT) and Δ*crp* derivative strains grown in LB at 37°C up to an OD_600nm_ of 0.4. The transcriptional expression is shown in relative values. In each case the reference (WT) value was set to one. Miller units in WT strains, *hilD*76-*lacZ* 63.3 +/- 5.9; *hilD*965-*lacZ* 5193.0 +/- 334.6; *hilD*1235-*lacZ* 38.3+/- 2.0. (B) Immunodetection of HilD-3xFLAG. Two different genetic constructs were used: one containing the *hilD* 3’UTR (+UTR) and the other lacking that region (-UTR). Immunodetection was assessed in whole cell extracts from cultures of the WT and Δ*crp* derivative strains grown as in A. (C) Immunodetection of the SPI-1 encoded SipA-3xFLAG protein was performed on whole cell extracts from two independent cultures of the WT and Δ*crp* derivative strains, either in the presence (+UTR) or the absence (-UTR) of the *hilD* 3’UTR. Cultures were grown as in A. In B and C, Coomassie Blue staining of the whole cell extracts serve as loading controls. Full length images of the Western blots, including molecular mass markers, are shown in S12 Fig. (D) *sipC* transcriptional expression was monitored in cultures grown as in A of the WT and Δ*crp* derivative strains carrying a *hilD* native gene or a derivative *hilD* lacking the 3’UTR. The transcriptional expression is shown in relative values. In each background (+UTR, -UTR) the activity reference of WT was set to one. Miller units in WT strains, UTR^+^ 73.6 +/- 11.8; UTR^-^ 13783.0 +/- 1142.5. In A and D, the β-galactosidase activity was determined for three independent cultures, average and standard deviation is shown. **, *p*< 0.01; ***, *p*< 0.001.

The *hilD* mRNA possess an unusually long (310 nt) 3’UTR that has an overall negative effect on *hilD* expression [11]. If the 3’UTR is deleted, the *hilD* mRNA accumulates and the SPI-1 genes are induced concomitantly [11]. Of note, the above-described effect of CRP-cAMP on *hilD* transcription (Fig 3C) was elucidated using a *hilD*-*lacZ* fusion at position +1,235 (relative to the transcription start site), containing the *hilD* coding sequence and the full-length 3’UTR. To determine whether the *hilD* 3’UTR is important in CRP-mediated regulation, a proximal fusion at position +76 was constructed. Remarkably, the Δ*crp* mutation had no effect on this proximal fusion (Fig 4A). This indicates that either CRP-cAMP does not regulate *hilD* expression at the level of transcription initiation or that the HilD protein is required for the induction of transcription initiation in a *crp-*deficient strain. To discriminate between these two possibilities, a *hilD*-*lacZ* fusion at position +965 was constructed, carrying the whole *hilD* coding sequence but lacking the *hilD* 3’UTR. As shown in Fig 4A, the Δ*crp* mutation did not lead to a significant induction even when the full coding sequence was included (*hilD*965-*lacZ*), as compared to a 6-fold induction detected using the *hilD*1235-*lacZ* fusion which includes both the *hilD* coding sequence and its 3’UTR. Although we cannot fully rule out a potential effect of CRP on *hilD* transcription, the different behavior of the *hilD*965-*lacZ* and *hilD*1235-*lacZ* reporters clearly points towards the 3’UTR being crucial for CRP-mediated post-transcriptional regulation of *hilD*.

The relevance of the 3’UTR in CRP-mediated regulation of HilD expression was supported by i) a Δ*crp*-dependent increase in the levels of HilD-3xFLAG protein was only detected when the *hilD*-3xFLAG mRNA contained the 3’UTR (Fig 4B) and ii) similarly, the Δ*crp*-dependent increase in SipA levels was only detected in strains carrying a *hilD* allele with the 3’UTR (Fig 4C). Additionally, the transcriptional expression of the SPI-1 gene *sipC* can be monitored as a proxy for HilD activity in the cell, since *sipC* upregulation in a *crp* mutant strain requires the presence of HilD (S4 Fig). Consistently, *sipC-lacZ* was upregulated in a Δ*crp* mutant background only when the *hilD* allele carried its native 3’UTR (Fig 4D). Our data also demonstrate that, according to the role attributed to the 3’UTR in the expression of *hilD* mRNA [11], there was an increase in the levels of HilD-3xFLAG, SipA-3xFLAG and *sipC*-*lacZ* when the 3’UTR was removed as compared to the parental strains carrying the native *hilD* mRNA containing the 3’UTR.

### A small RNA is involved in CRP-mediated regulation of *hilD*

**Fig 5:**
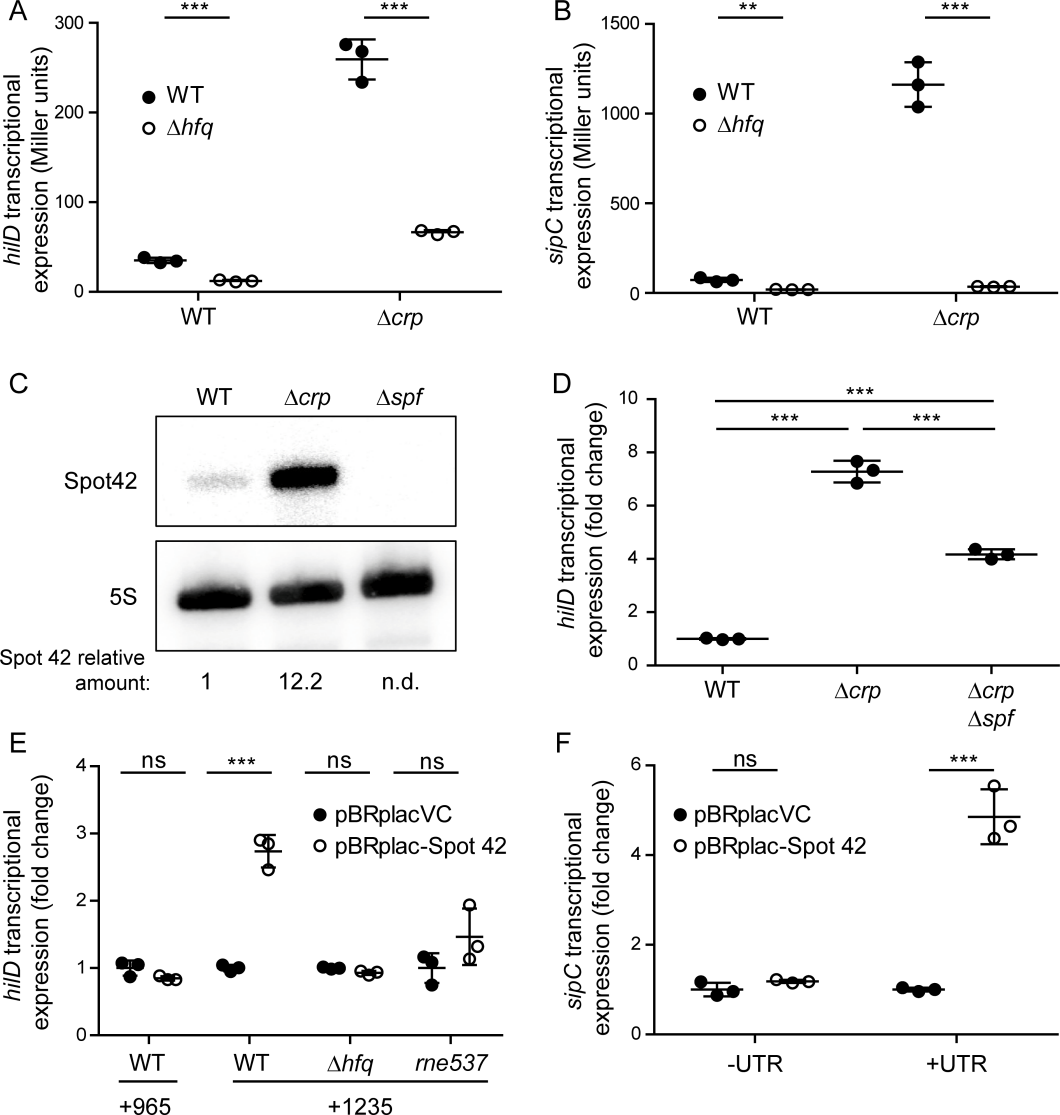
Spot 42 is under the control of CRP-cAMP in *Salmonella* and is involved in *hilD* regulation. *hilD* (*hilD*1235-*lacZ*) (A) and *sipC* (*sipC*-*lacZ*) (B) transcriptional expression was monitored in the wild-type (WT) and Δ*crp* derivative strains in either an *hfq*^+^ or Δ*hfq* genetic background. Cultures were grown in LB at 37°C up to an OD_600nm_ of 0.4. (C) Northern blot analysis for Spot 42 sRNA. Total RNA samples were extracted from cultures of the WT, Δ*crp* and Δ*spf* strains grown as in A. 5S rRNA was detected as loading control. For quantification, the ratio [Spot 42/5S] of three independent experiments was set to 1 in the wild-type strain. As a control, Spot 42 was not detected (n.d.) in a Spot 42 deficient strain. Full length images of the Northern blots, including molecular mass markers, are shown in S12 Fig. (D) *hilD* transcriptional expression was assessed in the WT, Δ*crp* and Δ*crp* Δ*spf* derivative strains carrying a *hilD*1235-*lacZ* chromosomal fusion (+UTR). The transcriptional expression is presented in relative values, the reference value (WT) was set to one. Miller units WT *hilD*1235-*lacZ*, 40.6 +/- 1.3. (E) *hilD* transcriptional expression was assessed upon over-expression of Spot 42 (pBRplac-Spot 42). β-galactosidase activity was measured in WT strains carrying two different chromosomal transcriptional fusions: the *hilD*965-*lacZ* (lacking the 3’UTR) and the *hilD*1235-*lacZ* fusion (containing it). The transcriptional activity of *hilD*1235-*lacZ* was additionally assessed in both Δ*hfq* and *rne537* derivative strains. The transcriptional expression is shown as relative values; the reference values (from strains containing the pBRplacVC) were set to one. Miller units for pBRplacVC *hilD*965-*lacZ* 4381.6 +/- 490.3; for *hilD*1235-*lacZ* WT pBRplacVC 33.6 +/- 1.6, Δ*hfq* pBRplacVC 12.1 +/- 0.1 and *rne*537 pBRplacVC 175.8 +/- 38.8. (F) *sipC* (*sipC*-*lacZ*) transcriptional expression was assessed in the WT strain upon over-expression of Spot 42 in presence (+UTR) or absence (-UTR) of the *hilD* 3’UTR. The transcriptional expression is presented as relative values. The reference values (from strains containing pBRplacVC) were set to one. Miller units in presence of (+UTR) 82.4 +/- 12.7, and in absence of (-UTR) 11967.1 +/- 507.0. In all cases, cultures were grown in LB at 37°C up to an OD_600nm_ of 0.4. β-galactosidase activity was measured from three independent cultures, averages and standard deviations are shown. **, *p*< 0.01; ***, *p*< 0.001; ns, not significant.

Based on the fact that CRP-cAMP is a transcriptional factor, it is surprising that the CRP-mediated regulation of HilD occurs at the post-transcriptional (and not the transcriptional) level, requiring the *hilD* 3’UTR. In other words, the data shown suggest that CRP-cAMP modulates *hilD* expression by an indirect mechanism. In line with previous reports [11,33], we found that the Δ*crp-*dependent activation of *hilD* expression, as judged by the *hilD*1235-*lacZ* fusion (containing the 3’UTR), was impaired in the absence of the sRNA chaperone Hfq (Fig 5A). Similarly, the drastic increase (16-fold) in *sipC* expression caused by the deletion of *crp* was abolished in the absence of Hfq (Fig 5B). We thus hypothesized that Hfq-dependent sRNA may be involved in the CRP-mediated regulation of *hilD*.

In search for candidate sRNAs in *Salmonella*, we focused on Spot 42 (encoded by the *spf* gene) which is transcriptionally controlled by CRP-cAMP in the closely related species, *E*. *coli* [34]. Work by the Storz and Valentin-Hansen laboratories had established this sRNA to be a general repressor of sugar-related mRNAs during CRP-mediated catabolite repression [23]. In *Salmonella*, Spot 42 is highly abundant, with maximal expression in mid-logarithmic phase and reduced upon entry into stationary phase [35]. We tested by Northern blot whether Spot 42 is under CRP-cAMP control also in *Salmonella* (Fig 5C). In the mid-logarithmic growth phase a 12-fold upregulation of Spot 42 sRNA was detected in the CRP-deficient compared to the wild-type strain. This pattern was further validated using a chromosomal *spf*-*lacZ* fusion (S5 Fig). Additionally, *spf* expression was assessed at early stationary phase. Interestingly, the *spf* induction detected in the Δ*crp* derivative strain in mid-logarithmic phase was no longer observed in early stationary phase (S5 Fig), reflecting the divergent effects observed for CRP on SPI-1 expression in exponential versus early stationary phase.

To establish whether Spot 42 is involved in the CRP-cAMP-mediated regulation of *hilD*, expression studies in strains either deficient in Spot 42 or over-expressing the sRNA were performed. In the absence of CRP, a partial but significant drop in the upregulation of *hilD* in the Spot 42-deficient background (Δ*spf*) was detected (Fig 5D). In contrast, ectopically expressing Spot 42 stimulated *hilD* expression. This suggests that Spot 42 is indeed involved in CRP-mediated repression of *hilD*. Importantly, over-expression of Spot 42 caused a 3-fold increase in *hilD* expression only when the 3’UTR was present, which implicates the *hilD* 3’UTR as a previously unknown target of this sRNA (Fig 5E). Consistently, a 2.5-fold increase in HilD-3xFLAG levels was detected upon the over-expression of Spot 42 (S6 Fig).

In agreement with previous data (Fig 5A, [29,35,36]), the positive effect of Spot 42 on *hilD* requires the chaperone Hfq (Fig 5E). As it has been shown before [29], Hfq binds to both Spot 42 and the *hilD* 3’UTR (S7 Fig). In addition, the major endoribonuclease RNase E has been suggested to play a role in 3’UTR mediated silencing of *hilD* expression [11]. Accordingly, *hilD* induction upon over-expression of Spot 42 was partially lost in the *rne537* background encoding an RNase E with a truncated C-terminal domain that is defective in degradosome assembly [37] (Fig 5E). These results suggest that both Hfq and RNase E are involved in the Spot 42-mediated effect on *hilD* expression.

The involvement of Spot 42 in the control of SPI-1 gene expression was further assessed by examining the transcriptional activity of a *sipC*-*lacZ* reporter. Transcription was monitored in either strains carrying the native *hilD* (+UTR) or strains from which the *hilD* 3’UTR had been removed (-UTR). As shown in Fig 5F, there was a 5-fold induction of *sipC*-*lacZ* upon over-expression of Spot 42 in the +UTR background, whereas *sipC* transcription was unaffected when Spot 42 was over-expressed in a background lacking the *hilD* 3’UTR (-UTR).

To confirm that the *hilD* 3’UTR is targeted by Spot 42, the *hilD* 3’UTR was cloned downstream of the *gfp* coding sequence expressed from a constitutive promoter. Expression of this genetic reporter was monitored in either the presence or absence of Spot 42. Co-expression of the sRNA led to a nearly two-fold increase in fluorescence, suggesting that Spot 42 targets the *hilD* 3’UTR regardless of the genomic location of the latter (S8 Fig). Overall, these results led us to conclude that Spot 42 sRNA activates *hilD* expression (either directly or indirectly) in a manner that requires the presence of the *hilD* 3’UTR.

### The unstructured region III of Spot 42 is required for *hilD* regulation

**Fig 6:**
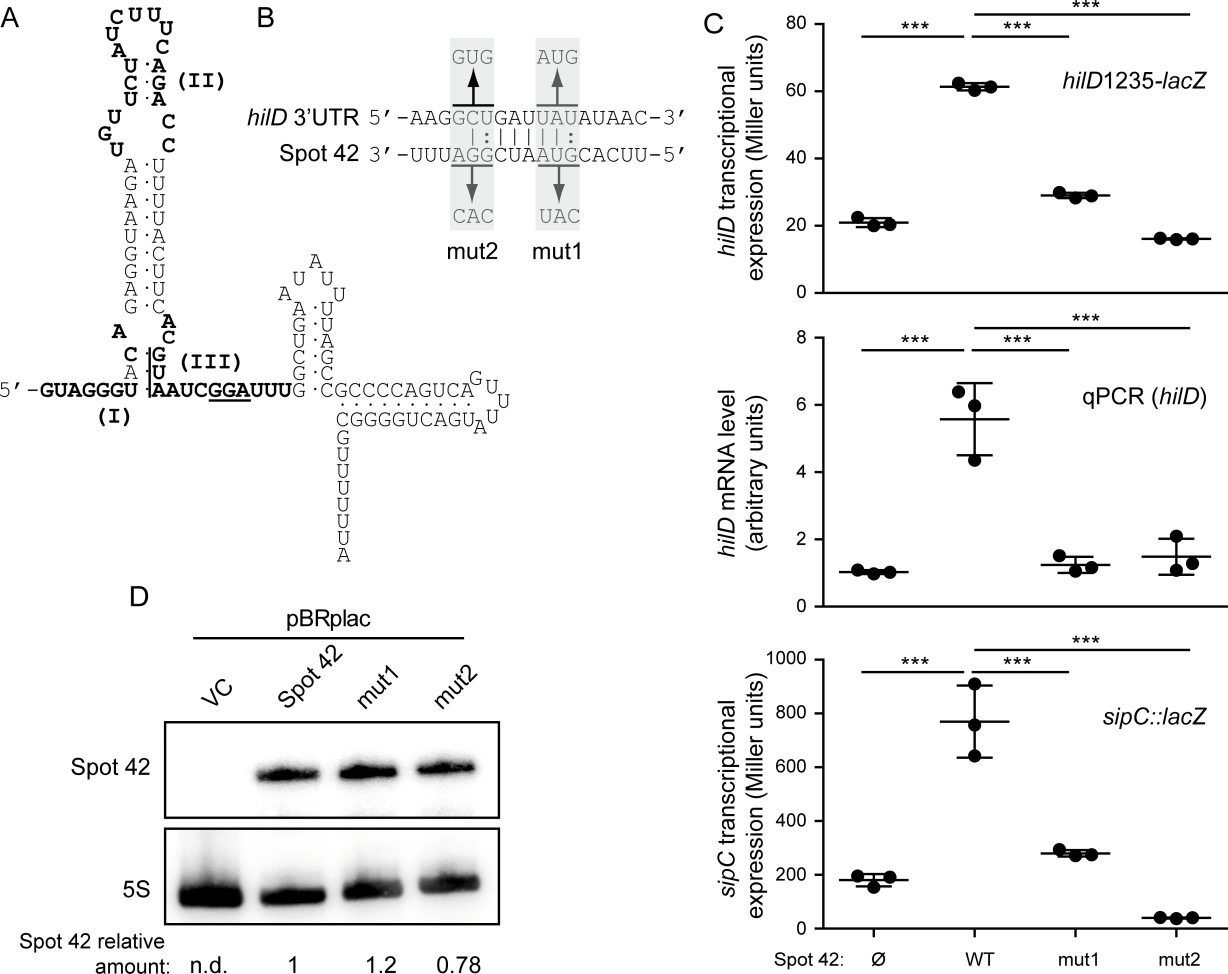
Unstructured region III is required for Spot 42 mediated stimulation of *hilD* expression. (A) Secondary structure prediction of Spot 42. The three unstructured regions (I, II, and III) are highlighted. The figure was adapted from [24]. (B) Putative interaction site between *hilD* 3’UTR and the unstructured region III of Spot 42 as predicted by IntaRNA software. The full *spf* sequence encoding for Spot 42 sRNA was used as an input sRNA sequence, and the 310 nt of the *hilD* 3’UTR were used as a target RNA sequence. The predicted base pairing and the altered nucleotides in the mut1 and mut2 are underlined and sequence substitutions are indicated. (C) Transcriptional expression of *hilD* and *sipC* was assessed upon over-expression of Spot 42^WT^, Spot 42^mut1^ or Spot 42^mut2^. As a control, strains carrying the empty vector pBRplac (Ø) were included. For monitoring *hilD*1235-*lacZ* and *sipC*-*lacZ* expression, β-galactosidase activity was determined. For relative *hilD* mRNA quantification, qRT-PCR was performed and the expression levels of strains carrying the pBRplac empty vector were set to 1. Detection of *gapA* (GAPDH) was used as an internal control (see Material and Methods). In all cases data correspond to the average and standard deviation of three independent experiments. ***, *p*< 0.001. (D) Northern blot analysis for Spot 42 sRNA and its derivative mutants mut1 and mut2. Total RNA samples were extracted from cultures of the Δ*spf* strain carrying the control vector pBRplacVC or derivatives to over-express the different Spot 42 variants. 5S rRNA served as loading control. Full length images of the Northern blots, including molecular mass markers, are shown in S12 Fig. In C and D cultures were grown in LB at 37°C up to an OD_600nm_ of 0.4.

Spot 42 from *E*. *coli* and *Salmonella* share 98% sequence identity. Three unstructured regions (denoted I, II and III, Fig 6A) of Spot 42 have been identified in *E*. *coli* to participate in gene regulation through base-pairing interactions [24]. To dissect the mechanism of action of Spot 42 on *hilD* expression, we determined if specific regions within the sRNA were essential for regulation. The software IntaRNA [38], developed to search for putative interaction sites between two given RNA molecules, predicted an interaction between unstructured region III of Spot 42 and positions 1,129–1,138 of the *hilD* mRNA (i.e. a region within the 3’UTR). To test whether this unstructured region III of Spot 42 is required for the regulation of SPI-1 genes, two Spot 42 mutant variants were generated, *spf*-mut1 and *spf*-mut2 (Fig 6B). Over-expression of these Spot 42 derivatives was performed in strains carrying a deletion of the endogenous *spf* gene, and their effect on *hilD* expression was monitored by determination of i) *hilD*1235-*lacZ* expression, ii) *hilD* mRNA levels by qRT-PCR, and iii) *sipC*-*lacZ* expression as a readout for HilD activity.

In accordance with our previous results, over-expression of wild-type Spot 42 (Spot 42^WT^) upregulated *hilD*1235-*lacZ* and *sipC*-*lacZ* expression. Likewise, relative *hilD* mRNA levels were elevated upon Spot 42^WT^ over-expression (Fig 6C). Conversely, over-expression of neither Spot 42^mut1^ (*spf*-mut1) nor Spot 42^mut2^ (*spf*-mut2) induced *hilD*1235-*lacZ* expression, *hilD* mRNA levels or *sipC* expression, demonstrating that mutations in region III disrupt the stimulatory effect of Spot 42 on *hilD* expression. (Fig 6C). The substitutions introduced to generate *spf*-mut1 (GUA-CAU) and *spf*-mut2 (GGA-CAC) have previously been described in *E*. *coli*, where those substitutions were shown to retain Spot 42 steady-state levels [37,38]. Similarly, Northern blots showed that these mutations did not dramatically affect Spot 42 stability in *Salmonella* either (Fig 6D), arguing that the reduced capability of the Spot 42 mutant variants to induce SPI-1 was not due to lowered sRNA levels. Taken together, the results indicate that the unstructured region III of Spot 42 is required for the regulation of *hilD* expression.

The *in silico* prediction suggests that region III of Spot 42 interacts within the *hilD* 3’UTR, between positions 1,129 and 1,138 of the *hilD* mRNA. Accordingly, we generated two chromosomal compensatory mutations in the *hilD* 3’UTR that restore the base pairing of Spot 42^mut1^ or Spot 42^mut2^ with the putative target sequence within *hilD*. The mutant alleles were designated *hilD* 3’UTR^mut1^ and *hilD* 3’UTR^mut2^, respectively (Fig 6B). *sipC* expression was used as a readout for HilD activity. Over-expression of Spot 42^WT^ in both *hilD* 3’UTR^mut1^ and *hilD* 3’UTR^mut2^ genetic backgrounds induced expression of *sipC*-*lacZ*, indicating that substitution of those residues within the *hilD* 3’UTR did not impair the positive effect of Spot 42 on SPI-1 expression. Additionally, over-expression of either Spot 42^mut1^ or Spot 42^mut2^ in both *hilD* 3’UTR^mut1^ and *hilD* 3’UTR^mut2^ backgrounds did not reestablish the ability to induce *sipC* expression (S9 Fig). Despite unstructured region III of Spot 42 being responsible for *hilD* activation, these results suggest that the *in silico* predicted interaction site—positions 1,129–1,138 within *hilD* mRNA—is not the actual target site or, at least, not the unique interaction site with Spot 42. More complex interaction mechanisms cannot be ruled out such as multiple interactions sites of Spot 42 within the *hilD* 3’UTR.

### Spot 42 targets the last 185 nt of the *hilD* 3’UTR

**Fig 7:**
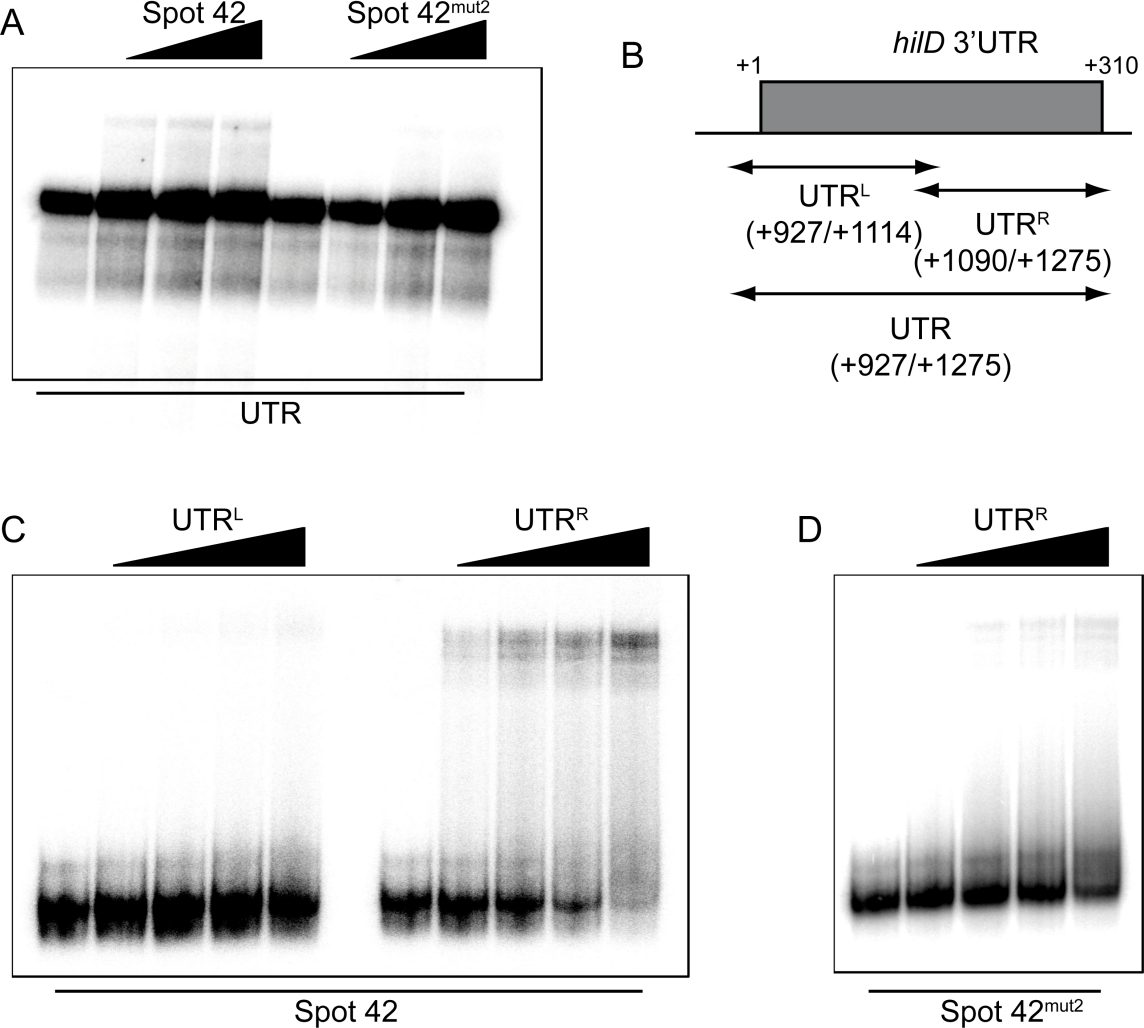
Spot 42 interacts with the downstream part of the *hilD* 3’UTR. (A) EMSA assay using 4 nM of *hilD* 3’UTR RNA radiolabeled incubated with increasing concentration of either Spot 42 or Spot 42^mut2^ RNA (0, 280, 560, 1700 nM). (B) Schematic representation of the *hilD* 3’UTR and the UTR^L^ and UTR^R^ fragments. EMSA using 4 nM of radiolabeled Spot 42^WT^ (C) or Spot 42^mut2^ (D) incubated with increasing concentrations (0, 56, 280, 560, 1,700 nM) of UTR^L^ or UTR^R^. All RNA transcripts used were obtained by T7 *in vitro* transcription. Samples were subjected to electrophoresis in a native gel and band shifts were observed upon drying and exposure of the gel.

Biochemical approaches were used to confirm the physical interaction between Spot 42 and the *hilD* 3’UTR. The ability of Spot 42 to bind to the *hilD* 3’UTR-derived fragments was assessed by electrophoretic mobility shift assays (EMSAs). EMSAs of radiolabeled full-length *hilD* 3’UTR incubated with increasing concentrations of Spot 42 confirmed a direct interaction between the two RNA species (Fig 7A). Consistent with our *in vivo* data, the unstructured region III of Spot 42 is required for the interaction with the *hilD* 3’UTR, since the binding affinity of the mutant version of Spot 42 (*spf*-mut2) was markedly reduced. Next, the *hilD* 3’UTR was divided into two halves, UTR^L^ and UTR^R^ (Fig 7B). The UTR^L^ fragment spans positions +927 to +1114 of the *hilD* mRNA (roughly the first half of the *hilD* 3’UTR), while UTR^R^ covers the second half of it (position +1090 to +1275) and includes the putative interaction site with the unstructured region III of Spot 42 (Fig 6B) as well as two Hfq binding sites as inferred from CLIP-seq [29]. EMSAs with radiolabelled Spot 42 and increasing concentrations of either one of the two UTR fragments were conducted. A concentration dependent upshift of Spot 42 was only observed upon addition of the UTR^R^ fragment with an apparent Kd of 80 nM but not upon addition of the UTR^L^ fragment (Fig 7C), indicating that Spot 42 interacts with the second half of *hilD* 3’UTR. Again, Spot 42-UTR^R^ interaction was specific as the affinity of the UTR^R^ fragment to the mutant version of Spot 42 (*spf*-mut2) was markedly reduced (Fig 7D). Further supporting this notion, in the reverse experiment increasing concentrations of Spot 42 did not lead to a band shift of the UTR^L^ but only of the UTR^R^ fragment (S10 Fig). Our results indicate that the loss of *hilD* activation by Spot 42^mut2^ *in vivo* is due to the inability of this mutant sRNA version to directly interact with the *hilD* mRNA, specifically with the second half of its 3’UTR. Overall, this makes Spot 42 the first Hfq-associated sRNA that potentially activates a *trans*-encoded target gene via its 3’UTR.

## Discussion

During the infection process, *Salmonella* relies on the expression of genes encoded on SPI-1 for epithelial cell invasion. Although SPI-1 is therefore crucial for *Salmonella* infection, it has a retarding effect on the growth rate, presumably as a consequence of the energetically high costs to produce the SPI-1 T3SS [3]. Accordingly, the expression of SPI-1 genes is tightly regulated [1]. Most relevant studies have focused on the regulatory pathways dedicated to induce SPI-1 under permissive conditions. However, as SPI-1 expression affects cell fitness, SPI-1 silencing mechanisms under non-permissive conditions, for instance in fast growing cells in the mid-logarithmic phase, are equally important. In this study, we identified CRP-cAMP, a metabolic sensor and global transcription factor [14,39], as a key player in the repression of SPI-1. CRP-cAMP is involved in a post-transcriptional regulatory circuit, controlling the expression of *hilD* by a mechanism dependent on its 3’UTR.

Coordination of metabolism and stress-related functions is crucial for the evolutionary success of bacterial populations. In pathogenic bacteria, the cross regulation between virulence factors, which can be considered within-host stress-related factors, and physiology is crucial for efficient colonization. A sudden shift between the expression of genes involved in active growth and genes involved in adaptation to stress might be required for rapid adaptation to changing conditions during the infection process. Secondary messengers such as cAMP, the intracellular levels of which can be altered by the action of both synthetases (adenylate cyclases) and degrading enzymes (phosphodiesterases), provide a rapid response system that can promote rapid changes in the expression profile. Although cAMP has traditionally been described as a regulator of metabolism, its role in the modulation of virulence-related functions has been extensively studied in several pathogens [40]. In *E*. *coli*, CRP-cAMP has been described to repress type 1 fimbriae expression during logarithmic growth [41]. Other secondary messengers, such as ppGpp, have also been reported to participate in the interplay between cell metabolism and virulence control [42]. Post-transcriptional regulation by small non-coding RNA confers to the cell another level for a rapid response to environmental conditions, in fact, a number of sRNAs have been found to play a relevant role in the metabolism-virulence crosstalk [43,44].

In this study we found that CRP-cAMP represses SPI-1 expression by modulating the expression of the regulator HilD (Figs 1–3). The role of HilD is not restricted to SPI-1; rather there is a complex cross-talk between HilD and master regulators of other virulence associated pathways. For example, it has been shown that HilD activates, under certain conditions, SPI-2 expression that is required for survival within macrophages [6,45]. Within macrophage-like cells, SPI-1 genes are downregulated and SPI-2 genes are induced [46]. CRP-cAMP is a regulator tailored to mediate rapid responses to environmental changes and may therefore be relevant for HilD-mediated regulation of virulence in response to the environmental conditions that *Salmonella* encounters through the infection process.

The CRP-mediated regulation of *hilD* does not occur at the transcriptional initiation level (Fig 4). Rather CRP-cAMP modulates *hilD* expression at the post-transcriptional level through the long 3’UTR (310 nt) of *hilD*. Post-transcriptional regulation is an extensively used mechanism to finely regulate virulence in bacterial pathogens [47,48]. The role of 5’UTRs in post-transcriptional gene expression control has been established and it is noteworthy that mRNAs of important SPI-1 regulators, such as *invF*, *hilA* and *hilE*, all carry long 5’UTRs [43,49–51]. In contrast, the involvement of 3’UTRs in post-transcriptional regulation is still poorly understood. Recently, it has been reported in *Staphylococcus aureus* that one-third of the cellular transcripts carry 3’UTRs longer than 100 nt [52]. In addition to be targets of regulation, 3’UTRs may provide regulators themselves, namely 3’UTR-derived sRNAs [36,53].

The 3’UTR of *hilD* constitutes a silencing module, since its deletion causes significant *hilD* upregulation. Although we are yet to elucidate the full molecular mechanism, the observed Hfq dependency suggested that one or several sRNAs are targeting the *hilD* 3’UTR [11]. Likewise, the CRP-mediated repression of *hilD* requires both the presence of the *hilD* 3’UTR and Hfq, indicating that CRP regulates *hilD* expression in an sRNA-mediated manner (Fig 4 and Fig 5).

Spot 42 is an integral member of the CRP-mediated gene expression network in *E*. *coli* [23–27] and its expression is repressed by CRP-cAMP in both *E*. *coli* and *Salmonella* ([47], Fig 5). Here we found that Spot 42 is involved in the CRP-mediated regulation of *hilD* expression, since the derepression of *hilD* in a Δ*crp* strain was diminished in the absence of Spot 42 and over-expressing Spot 42 caused a concomitant upregulation of *hilD* expression. Remarkably, Spot 42-mediated regulation targets the long *hilD* 3’UTR. The fact that the absence of Spot 42 did not completely abolish the *hilD* deregulation caused by the Δ*crp* mutation points at additional factors that could be involved in the described regulation. Although the nature of these factors remains fully elusive, it should be noted that these putative factors seem to also act through the *hilD* 3’UTR. Further studies will be required to determine whether other sRNAs or proteins plays a role in the CRP-mediated repression of *hilD* expression.

Both genetic and biochemical approaches point towards a direct interaction between Spot 42 and the *hilD* 3’UTR, involving the unstructured region III of Spot 42 (Figs 6 and 7). Although the exact target sequence within the *hilD* 3’UTR have not been identified, EMSA experiments indicate that the interaction occurs between Spot 42 and the downstream half of the 3’UTR (last 185 nt). This interaction is strongly diminished when the unstructured region III of Spot 42 is altered by base substitution in three positions previously described to be involved in base-pairing [23]. The recent finding that the transcription elongation factors GreA and GreB target the *hilD* 3’UTR to regulate *hilD* at permissive conditions [54] led us to speculate that transcriptional pausing might trigger a specific folding of the *hilD* 3’UTR important for post-transcriptional regulation. Overall, the regulation through the *hilD* 3’UTR seem to be complex and presumably several factors target the *hilD* 3’UTR. Although it has been proposed that intrinsic motifs in the long 3’UTR of *hilD* might confer susceptibility to degradation in a polynucleotide phosphorylase (PNP) and RNase E dependent manner, no effect in the stability of the *hilD* mRNA was detected [11]. Consistent with these data, we found no difference in the stability of the *hilD* mRNA between the wild-type and Δ*crp* strain in mid-logarithmic phase (S11 Fig). The exact mechanism by which these factors converge to regulate *hilD* expression should be the focus of future studies. Our results highlight the 3’UTR of *hilD* as a central hub in SPI-1 regulation and indicate that the whole *hilD* 3’UTR is required for the post-transcriptional regulation of *hilD*.

To our knowledge, there are no other examples of trans-encoded sRNAs targeting 3’UTRs. Of note, the cis-encoded sRNA GadY, which is encoded on the opposite strand of *gadX* 3’UTR, seems to positively regulate *gadX* through interaction with the *gadX*-*gadW* intergenic region [55]. Unlike Spot 42 and *hilD* which are expressed from regions in the chromosome over 1 Mb apart, GadY and *gadX* physically overlap. Global screens for Hfq-mediated sRNA-mRNA interactions [56,57] suggest, however, that 3’UTR targeting may be more common than currently appreciated.

**Fig 8:**
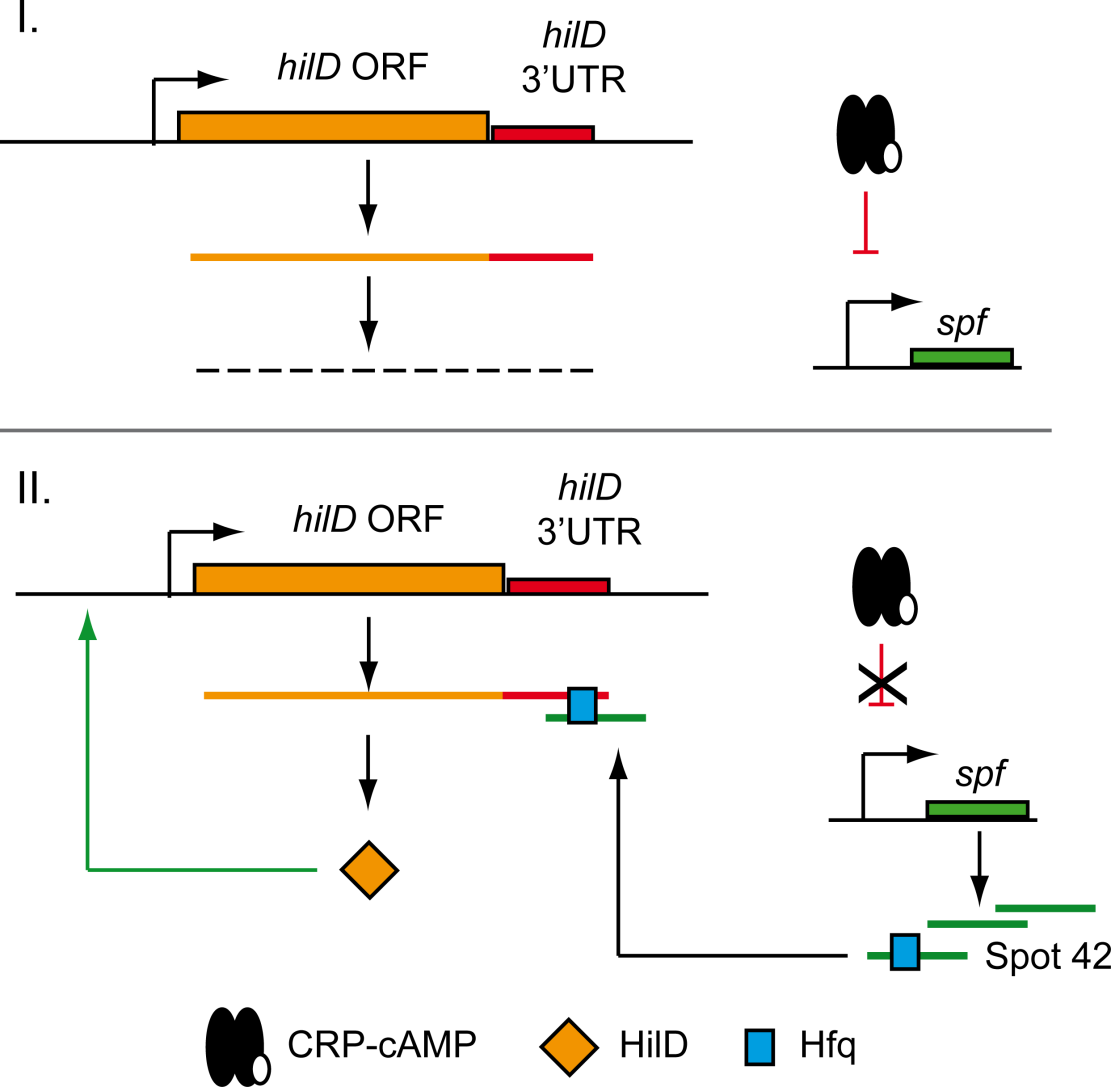
Proposed model of CRP-cAMP-mediated repression of *hilD* expression. I. Silencing of SPI genes (non-permissive SPI-1 conditions); II. Transition to SPI expression is triggered by Spot 42-mediated stimulation of *hilD* mRNA. Green and red lines indicate stimulation and repression, respectively.

In conclusion, our findings imply a novel mechanism in the complex regulatory network of SPI-1 expression. Under non-permissive conditions, very low transcriptional expression from the *hilD* gene occurs. Additionally, CRP-cAMP represses the transcription of the sRNA Spot 42, thereby maintaining basal levels of *hilD* expression. Consequently, despite *hilD* transcription occurs, only low levels of HilD protein arise (Fig 8 panel I). In contrast, environmental and/or physiological signals may relieve CRP-dependent Spot 42 repression. Upon binding to its 3’UTR in an Hfq-dependent manner, Spot 42 may exerts a positive effect on *hilD* mRNA, thereby activating HilD protein expression. As HilD auto-activates itself by promoting its own transcription, expression of some copies of HilD protein would likely be sufficient to amplify the final output (Fig 8 panel II). Thus, CRP-cAMP seems to play a relevant role by coordinating post-transcriptional virulence control in *Salmonella*. Somewhat similar to the described Spot 42-mediated regulation of SPI-1, the sRNA PinT acts as a timer of virulence gene expression in *Salmonella*, regulating SPI-2 genes through the modulation of CRP-cAMP [58]. Altogether, this highlights the emerging importance of collaborative activities of general transcription factors and sRNAs to precisely adjust the costly expression of major virulence factors to internal and external metabolic cues [44].

## Materials and methods

### Bacterial strains, plasmids and growth conditions

The bacterial strains and plasmids used in this study are listed in S1 Table.

*Salmonella enterica* serovar Typhimurium SL1344 and derivative strains were cultivated either in Luria Bertani broth (tryptone 10 g/l, yeast extract 5 g/l and sodium chloride 10 g/l). When required, the media was supplemented with ampicillin (Amp) 100 μg/ml, kanamycin (Km) 50 μg/ml, chloramphenicol (Cm) 15 μg/ml, or tetracycline (Tc) 15 μg/ml. Induction of genes cloned into pTRc99a was achieved by adding 0.1 mM IPTG.

Liquid cultures (20 ml of LB in 100 ml culture flasks) were inoculated to an OD_600nm_ of 0.001 and incubated at 37°C with vigorous shaking (200 rpm). An OD_600nm_ of 0.3–0.4 was considered mid-logarithmic phase of growth, while an OD_600nm_ of 2.0 was considered early stationary phase of growth.

### Genetic manipulations

The *cpdA* (SL1344_3157) gene was cloned into the IPTG inducible vector pTRc99a [59]. The *cpdA* coding sequence was PCR amplified by Phusion polymerase (Invitrogen) with the primers *cpdA*_*Xba*I_Fw and *cpdA*_*Sal*I6xHis_rev (S2 Table), subsequently digested with *Xba*I and *Sal*I and ligated into *Xba*I/*Sal*I digested pTRc99a.

The *spf* gene encoding Spot 42 sRNA was cloned into pBRplac vector [60]. *spf* was PCR amplified with the primers *spf*_*Aat*II_Fw and *spf*_*Eco*RI_rev (S2 Table), subsequently digested with *Aat*II and *Eco*RI and ligated into *Aat*II/*Eco*RI digested pBRplac. Mutations in Spot 42 (*spf*-mut1 and *spf*-mut2) were generated by assembly PCR and subsequent cloning in the pBRplac vector.

A *gfp-hilD* 3’ UTR construct was cloned in the backbone of plasmid pXG1 [61]. The *hilD* 3’UTR region was fused to *gfp* by overlapping PCR using chromosomal SV5015 and plasmid pXG1 as templates and the primers *gfp*_*Nhe*I_Fw, *gfp*_*hilD*_rev, *hilD_gfp*_Fw and *hilD_Xba*I_rev (S2 Table). The purified PCR fragment was subsequently digested with *Xba*I/*Nhe*I and ligated into an *Xba*I/*Nhe*I digested pXG1 vector, resulting in the plasmid pXG1 *gfp*-3’UTR.

Deletion mutants were generated by gene replacement as described by Datsenko and Wanner [62]. Briefly, antibiotic resistance cassettes carrying either Km^R^ or Cm^R^ resistance genes were amplified from pKD4 and pKD3, respectively. Primers used include a 40 bp sequence complementary to the region where the insertion was desired. Purified fragments were electroporated into strains carrying pKD46. Positive clones were selected in presence of the required antibiotic. When desired, the antibiotic resistance cassette was removed by expression of the Flp recombinase from the pCP20 plasmid, as described [63].

Deletion mutants, where the antibiotic cassette was removed, were further used for the generation of reporter gene fusions. Transcriptional *lacZ* fusions were generated as described [64], the remaining FRT-site was used to integrate plasmid pKG136.

Epitope tagged proteins were constructed as follows: HilA, HilD and SipA 3xFLAG tagged proteins were generated by a λRed recombinase system as described [65]. When desired, the Km^R^ cassette downstream of the 3xFLAG epitope was removed using the Flp recombinase (see above). In the HilD 3xFLAG construct retaining the Km^R^ cassette, the *hilD* coding sequence and 3’UTR are split and not co-transcribed. Thus, the Km^R^ cassette was removed, with the *hilD* 3’UTR now located right downstream of the 3xFLAG epitope. Oligonucleotides used to generate the constructs are listed in S2 Table. All strains were PCR confirmed and integrity of the sequence was checked by DNA sequencing.

Chromosomal modifications in the *hilD* 3’UTR region were generated by scarless mutation [66]. Briefly, a 1 kb fragment containing the *hilD* 3’UTR sequence was cloned into pGEM vector, desired point mutations were generated via the Quick change method using the *hilD*-UTR oligonucleotides listed in S2 Table. Subsequently, the vector was digested with *Sac*I/*Xba*I and ligated into the suicide plasmid pDMS197 [67]. The derivative pDMS197 was propagated in S17-1 lambda *pir* and used as donor in matings with SV5015 Δ*spf sipC*-*lacZ*. Trans-conjugants were selected for tetracycline resistance. Selected clones were grown in salt-free nutrient broth supplemented with 5% sucrose. Individual tetracycline-sensitive clones were checked by PCR and subsequent DNA sequencing to select the clones carrying the desired chromosomal mutation.

### Collecting protein extracts

To obtain whole cell and secreted protein extracts, LB cultures were grown at 37°C and processed as previously described [54]. Samples in Laemmli sample buffer were subjected to SDS-PAGE separation. Normalization of the loading samples was performed based on the culture biomass (OD_600nm_). Coomassie stain was used to visualize protein bands.

### Western blot assay

Protein extracts were subjected to SDS-PAGE separation, transfer to PVDF filter and subsequent immunodetection using monoclonal Anti-FLAG (Sigma), Anti-His (Sigma) or polyclonal Anti-SopE [68] as primary antibodies. Commercial polyclonal anti-mouse (Promega) and anti-rabbit (GE Healthcare) secondary antibodies conjugated to horseradish peroxidase were used. For detection, ECL Prime Western Blotting Detection Reagent (GE Healthcare) served as a substrate. Chemi-luminescence was detected using Chemidoc equipment (Bio-Rad). As a control prior to the immunodetection, all whole cell extract samples were analyzed by SDS-PAGE and Coomassie staining to ensure proper normalization of the loaded amounts.

### Protein identification

For protein identification, the protein bands from Coomassie stained SDS-PAGE gels were trypsin digested and analyzed by LC-MS/MS by the Proteomic facility from the Scientific Park of Barcelona (PCB).

### β-galactosidase assay

β-galactosidase activity was measured as described previously [69]. Activity determination was performed in technical duplicates for each of three biological replicates.

### RNA isolation

For each strain, samples from three independent LB cultures grown at 37°C to mid-logarithmic phase (OD_600nm_ 0.4) were processed. RNA was extracted by classical hot phenol method. RNA quality and concentration was assessed by an Agilent Technologies Bioanalyzer 2100.

### qRT-PCR

Quantitative reverse transcription-PCR (qRT-PCR) was performed as previously described [54]. The relative amount of target cDNA was normalized using the *gapA* (GAPDH) gene as an internal control. Oligonucleotides used for qRT-PCR are listed in S2 Table.

### Northern blot

Electrophoretic separation of total RNA samples was carried out in Tris-Borate-EDTA (TBE) 8% acrylamide gels containing 8.3 M urea. Samples were prepared by mixing 10 μl of RNA samples with 10 μl of urea dye (2x) loading buffer and incubated for 10 minutes at 65°C, immediately chilled on ice and loaded for electrophoretic separation at 30 mA for 2 hours.

RNAs were transferred to Hybond N+ (GE Healthcare) filters by semi-dry TBE based transfer for 2 hours at 400 mA. RNAs were subsequently fixed to the filter by UV crosslinking. Filters were then hybridized with radiolabeled oligos, sequences are given in S2 Table. Images of radioactive filters were obtained with the FLA-5100 imaging system (Fujifilm) and quantification was performed using Image J software.

### Electrophoretic mobility shift assay (EMSA)

RNA-RNA interactions were detected by Electrophoretic Mobility Shift Assay (EMSA) as described in [70]. First, DNA templates for *in vitro* T7 RNA transcription were generated by PCR, primers used are listed in S2 Table. RNA was produced *in vitro* by following the Megascript transcription procedure from Ambion. Then, either the sRNA (Spot 42) or the target RNAs (*hilD* 3’UTR, UTR^R^ or UTR^L^) was dephosphorylated and 5’ labeled with [(α-^32^P) ATP]. The putatively interacting RNAs were next incubated in structure buffer (Ambion): In a 10 μl final volume, 4 nM of the radiolabeled RNA was incubated with increasing concentrations of the unlabeled RNA (0, 56, 280, 560, 1700nM). Samples were incubated at 37°C for 1 hour and subjected to electrophoresis in a native 6% acrylamide gel. For specific RNA detection, acrylamide gels were dried and exposed. Images were obtained as for Northern blots.

### GFP measurement

For single-cell analysis, cell cultures were grown to the desired conditions, pelleted and resuspended in PBS. The bacterial suspensions were then fixed in 4% formaldehyde. The fluorescence of 20,000 bacterial cells was measured by flow cytometry using preset parameters for GFP (excitation wavelength of 484 nm and emission wavelength of 512 nm). Measurements were performed in technical duplicates for each three biological replicates; average was used to compare GFP expression.

### Invasion assay in HeLa epithelial cells

HeLa human epithelial cells (ATCC CCL2) were cultured in tissue culture medium (Dulbecco’s Modified Essential Medium (DMEM) supplemented with 10% fetal calf serum and 1mM glutamine). HeLa cells were seeded the day before the infection in 24-well plates containing 0.5 ml of DMEM per well and grown at 370°C, 5% CO2. Bacterial cells grown at 37°C to different phases of growth were prepared in DMEM. The bacterial mixture was added to HeLa cells to reach a multiplicity of infection (MOI) of 75 bacteria per eukaryotic cell. 30 minutes post-infection HeLa cells were washed twice with phosphate buffered saline (PBS) and incubated in fresh DMEM medium containing 100 μg/ml gentamicin for 90 minutes. Numbers of viable intracellular bacteria were obtained after lysis of infected cells with 1% Triton X-100, and subsequent plating. Infections were carried out in triplicate. Invasion rate is defined as the intracellular bacteria recovered versus viable bacteria used to infect the HeLa cells (initial inoculum). Invasion rates were normalized to bacterial culture of a wild type strain.

### Statistical analysis

GraphPad Prism 5.0 software was used for data analysis. Two-tailed Student’s *t*-test were carried out and p-values < 0.05 were considered significant.

## Supporting information

S1 Fig Overexpression of CpdA. Upper panel. Immunodetection of CpdA-6xHis was performed in extracts of the wild-type (WT) and a Δ*cya* derivative strain carrying either pTrc99a (-, control vector) or pCpdA (+, pTrc99a+*cpdA*). Cultures were grown in LB supplemented with IPTG (0.1 mM) at 37°C up to an OD_600nm_ of 0.4. The band corresponding to the CpdA protein is indicated with an arrowhead. Lower panel. Coomassie Blue staining of the whole cell extracts serve as loading controls. M: molecular mass markers (kDa).
(TIF)

S2 Fig Effect of Δ*hilA* mutation on the expression of SPI-1 encoded secreted effector proteins. Cell-free supernatants from two independent cultures of the WT and Δ*hilA* strain grown up to early stationary phase (OD_600nm_ 2.0) were TCA precipitated. The resulting extracts were analyzed by SDS-PAGE and Coomassie staining. Arrowheads indicate presumed secreted effector proteins from *Salmonella*. Size in kDa of molecular mass marker bands (M) are indicated.
(TIF)

S3 Fig Effect of Δ*crp* mutation on the *hilA* mRNA levels. Relative quantification by qRT-PCR of *hilA* mRNA in a Δ*crp* derivative strain compared to wild type (WT). The reference value (WT) was set as one. Detection of *gapA* (GAPDH) was used as an internal control (see Materials and Methods). RNA samples were extracted from cultures of the WT and Δ*crp* derivative strains grown in LB at 37°C up to an OD_600nm_ of 0.4. The average and standard deviation from three independent experiments are shown. *** *p*< 0.001.
(TIF)

S4 Fig The induction of *sipC* expression in the Δ*crp* derivative strain is strictly dependent on the presence of *hilD*. Transcriptional expression of *sipC*-*lacZ* was monitored in the wild type (WT) and Δ*crp* derivative strains in either a *hilD*^+^ or *hilD*^-^ genetic background. Cultures were grown in LB at 37°C up to an OD_600nm_ of 0.4. The β-galactosidase activity from three independent experiments was averaged and the standard deviation is shown. ***, *p*< 0.001; ns, not significant.
(TIF)

S5 Fig CRP-cAMP represses *spf* expression in exponentially growing cells. Transcriptional expression of *spf* in the wild type (WT) and Δ*crp* derivative strains was monitored by β-galactosidase activity determination of a *spf*-*lacZ* chromosomal fusion. LB cultures were grown at 37°C up to either mid-logarithmic (OD_600nm_ 0.4) or early stationary (OD_600nm_ 2.0) phase. Data from three independent experiments are averaged and the standard deviation is shown. ***, *p*< 0.001; ns, not significant.
(TIF)

S6 Fig Spot 42 stimulates HilD expression. **A.** Immunodetection of HilD-3xFLAG was performed on whole cell extracts from cultures of the wild type (WT) strain (+UTR) carrying either the pBRplacVC (control vector, reference value) or pBRplac-Spot 42. Coomassie Blue staining of the whole cell extracts serve as loading controls. **B**. Merged image of white light caption for detection of the molecular mass marker and the chemiluminiscence detected bands in an extract from WT carrying the pBRplacVC. Molecular mass markers in kDa. **C**. The band corresponding to HilD-3xFLAG (indicated with an arrowhead) is easily identified as the protein band over-accumulated in Δ*crp* as compared to WT.
(TIF)

S7 Fig Hfq binding assessment to *hilD* 3’UTR and Spot42 by EMSA. *In vitro* transcribed RNA was radiolabeled. 4 nM of the radiolabeled RNA was incubated with increasing concentration of purified Hfq (0, 1.3, 4, 13, 40, 130 nM) and subjected to electrophoresis in a native gel. Band shift was observed upon drying and exposure of the gel.
(TIF)

S8 Fig Spot 42-mediated regulation of a genetic construct carrying the *hilD* 3’UTR motif. GFP fluorescence assessment by flow cytometry of GFP-*hilD* 3’UTR upon overexpression of the sRNA Spot 42. Cultures of Δ*spf* strains carrying the construct pXG1*gfp*-*hilD*3’UTR in presence (pBRplac-Spot42) or absence of the sRNA Spot 42 (pBRplacVC) grown in LB at 37°C up to an OD_600nm_ of 0.4. Data from three independent experiments are averaged and the standard deviation is shown. ***, *p*< 0.001.
(TIF)

S9 Fig Compensatory chromosomal mutations within the *hilD* 3’UTR. Transcriptional expression of *sipC*-*lacZ* was monitored in two different *hilD* backgrounds: *hilD* 3’UTR^mut1^ and *hilD* 3’UTR^mut2^. *sipC-lacZ* expression was assessed upon overexpression of either Spot 42^WT^, Spot 42^mut1^ and Spot 42^mut2^. β-galactosidase activity was determined for three independent cultures, average and standard deviation are shown. ***, *p*< 0.001; ns, not significant. In all cases, bacterial cultures were grown in LB at 37°C up to an OD_600nm_ of 0.4.
(TIF)

S10 Fig Spot 42 interacts with the *hilD* 3’UTR. EMSA assay using 4 nM of either UTR^L^ or UTR^R^ fragments incubated with increasing concentration (0, 56, 280, 560, 1700 nM) of Spot 42 RNA. All RNA molecules used were obtained by T7 *in vitro* transcription. Samples were subjected to electrophoresis in a native gel and band shift was observed upon drying and exposure of the gel.
(TIF)

S11 Fig *hilD* mRNA stability. *hilD* mRNA was detected by Northern blot. Culture of wild type (WT) and Δ*crp* were grown to mid-logarithmic phase (OD_600nm_ 0.4), rifampicin was added (500 μg/ml) and samples were taken for total RNA extraction at 2, 4, 8, 16 and 32 min. Samples before rifampicin addition (time 0) were taken. RNA radiolabeled probe complementary to the first 300 nt of *hilD* mRNA was generated by *in vitro* T7 RNA transcription and used for *hilD* mRNA detection. tmRNA was detected as loading control. Full length image of the Northern blot is shown in S12 Fig.
(TIF)

S12 Fig Compendium of uncropped images used to generate Figs 1–6 and S11 Fig. (PDF)

S1 Table Strains and plasmids used in this work. (PDF)

S2 Table Oligonucleotides used in this work. (PDF)
