# Supplementary figures and images for: CRP-cAMP mediates silencing of Salmonella virulence at the post-transcriptional level
Source: PLoS Genet. 2018 Jun 7;14(6):e1007401. doi: 10.1371/journal.pgen.1007401 (PMC5991649; doi:10.1371/journal.pgen.1007401)

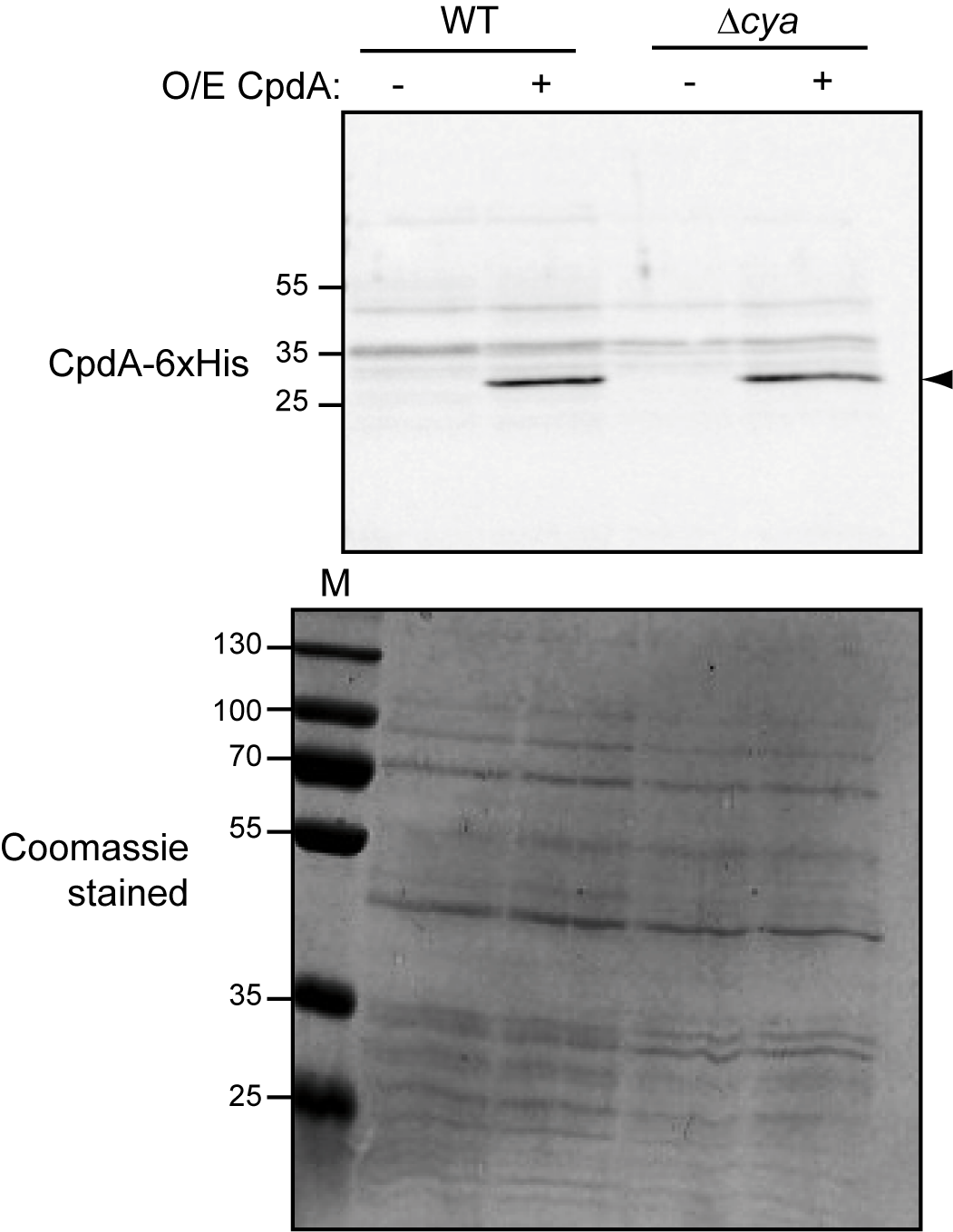

Supplement: S1 Fig — Upper panel. Immunodetection of CpdA-6xHis was performed in extracts of the wild-type (WT) and a Δcya derivative strain carrying either pTrc99a (-, control vector) or pCpdA (+, pTrc99a+cpdA). Cultures were grown in LB supplemented with IPTG (0.1 mM) at 37°C up to an OD600nm of 0.4. The band corresponding to the CpdA protein is indicated with an arrowhead. Lower panel. Coomassie Blue staining of the whole cell extracts serve as loading controls. M: molecular mass markers (kDa). (TIF) [file pgen.1007401.s001.tif]

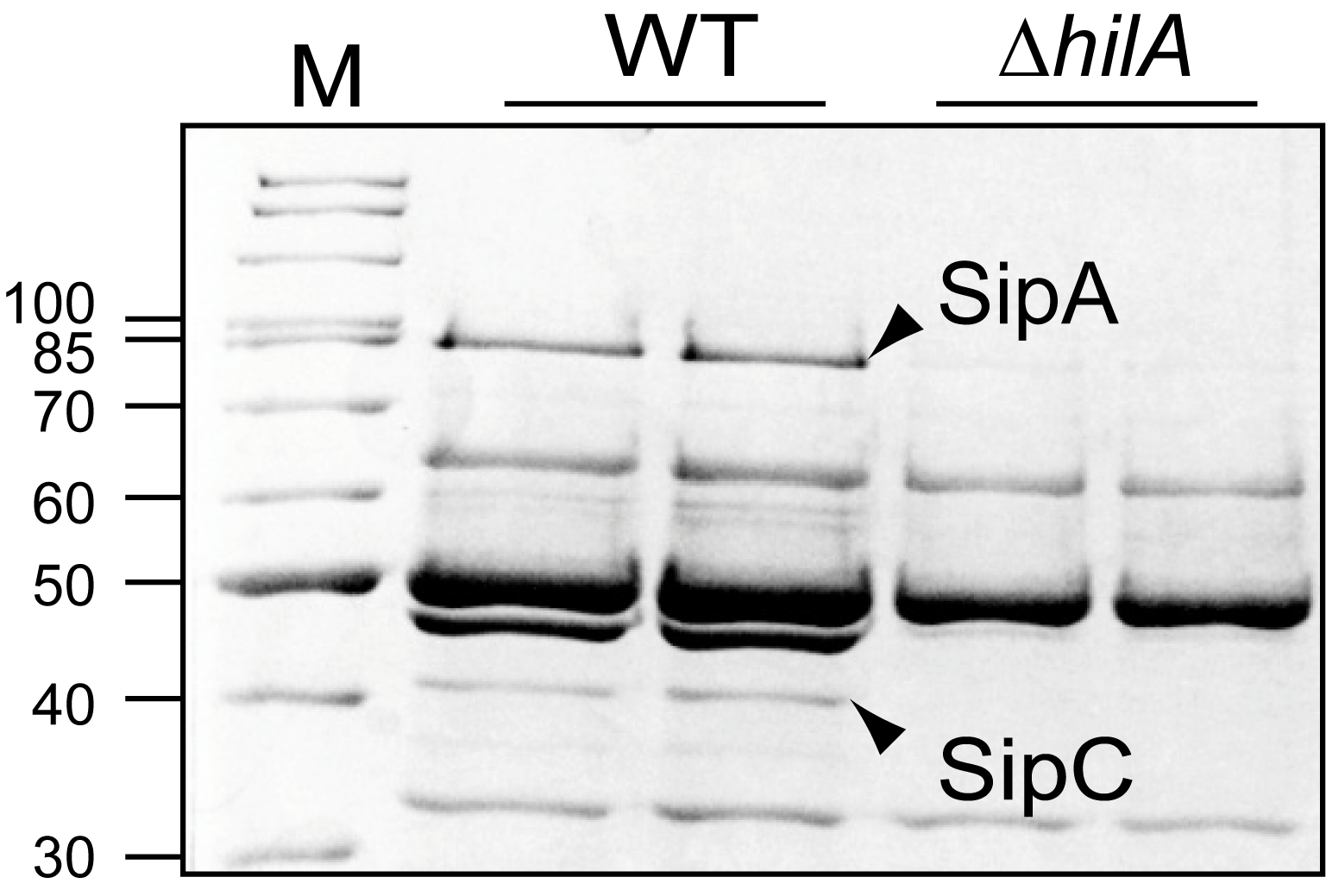

Supplement: S2 Fig — Cell-free supernatants from two independent cultures of the WT and ΔhilA strain grown up to early stationary phase (OD600nm 2.0) were TCA precipitated. The resulting extracts were analyzed by SDS-PAGE and Coomassie staining. Arrowheads indicate presumed secreted effector proteins from Salmonella. Size in kDa of molecular mass marker bands (M) are indicated. (TIF) [file pgen.1007401.s002.tif]

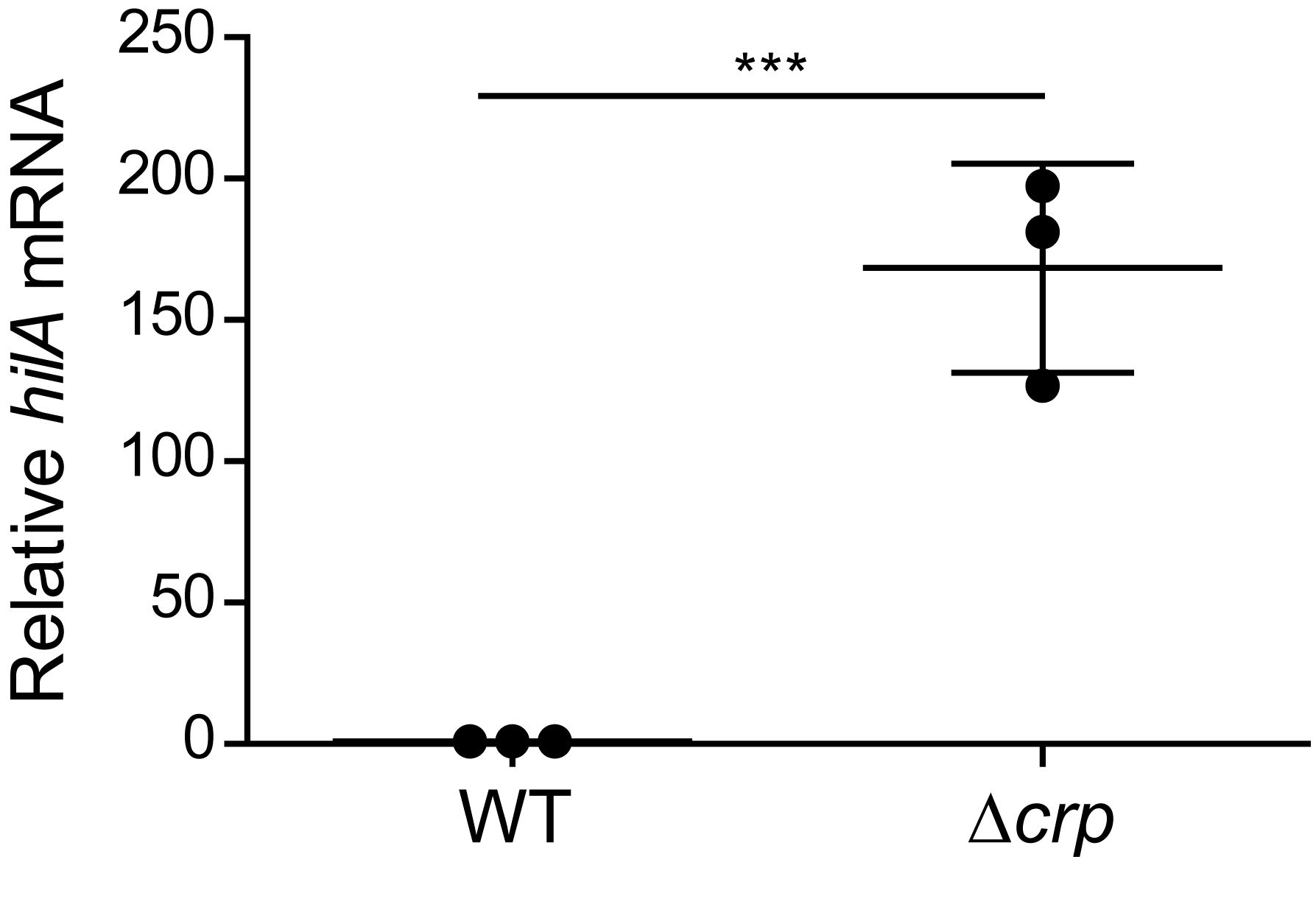

Supplement: S3 Fig — Relative quantification by qRT-PCR of hilA mRNA in a Δcrp derivative strain compared to wild type (WT). The reference value (WT) was set as one. Detection of gapA (GAPDH) was used as an internal control (see Materials and Methods). RNA samples were extracted from cultures of the WT and Δcrp derivative strains grown in LB at 37°C up to an OD600nm of 0.4. The average and standard deviation from three independent experiments are shown. *** p< 0.001. (TIF) [file pgen.1007401.s003.tif]

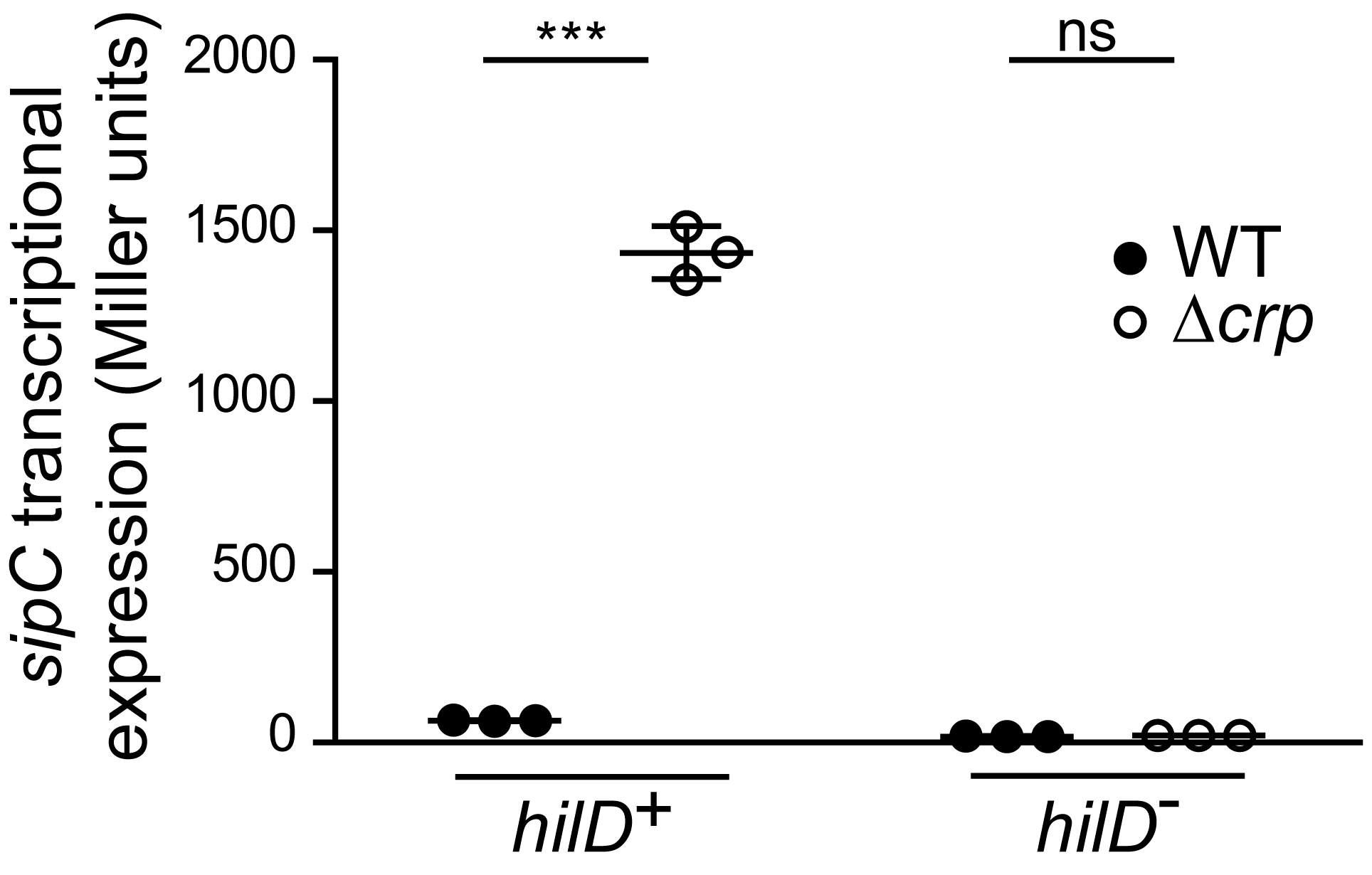

Supplement: S4 Fig — Transcriptional expression of sipC-lacZ was monitored in the wild type (WT) and Δcrp derivative strains in either a hilD+ or hilD- genetic background. Cultures were grown in LB at 37°C up to an OD600nm of 0.4. The β-galactosidase activity from three independent experiments was averaged and the standard deviation is shown. ***, p< 0.001; ns, not significant. (TIF) [file pgen.1007401.s004.tif]

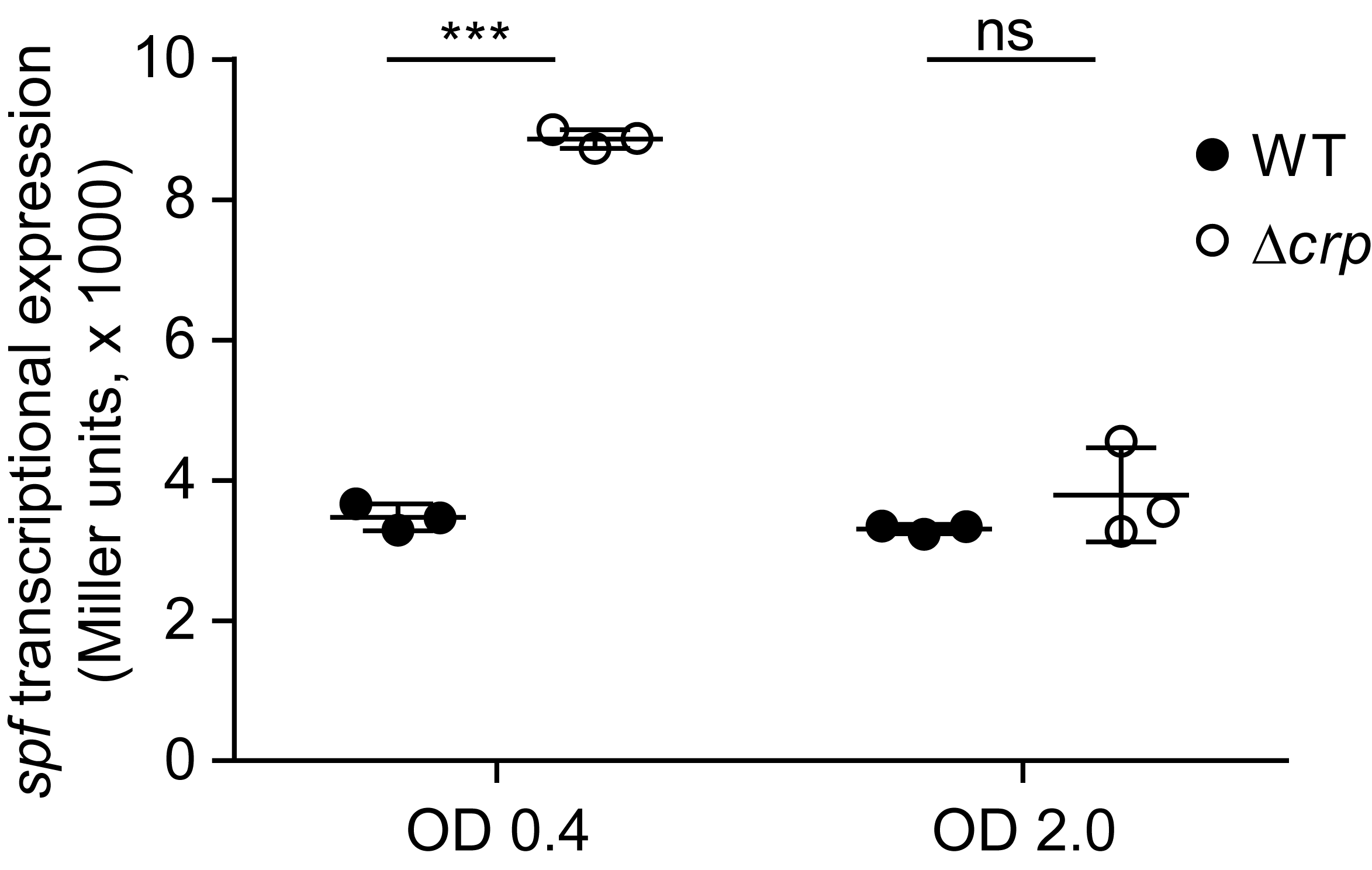

Supplement: S5 Fig — Transcriptional expression of spf in the wild type (WT) and Δcrp derivative strains was monitored by β-galactosidase activity determination of a spf-lacZ chromosomal fusion. LB cultures were grown at 37°C up to either mid-logarithmic (OD600nm 0.4) or early stationary (OD600nm 2.0) phase. Data from three independent experiments are averaged and the standard deviation is shown. ***, p< 0.001; ns, not significant. (TIF) [file pgen.1007401.s005.tif]

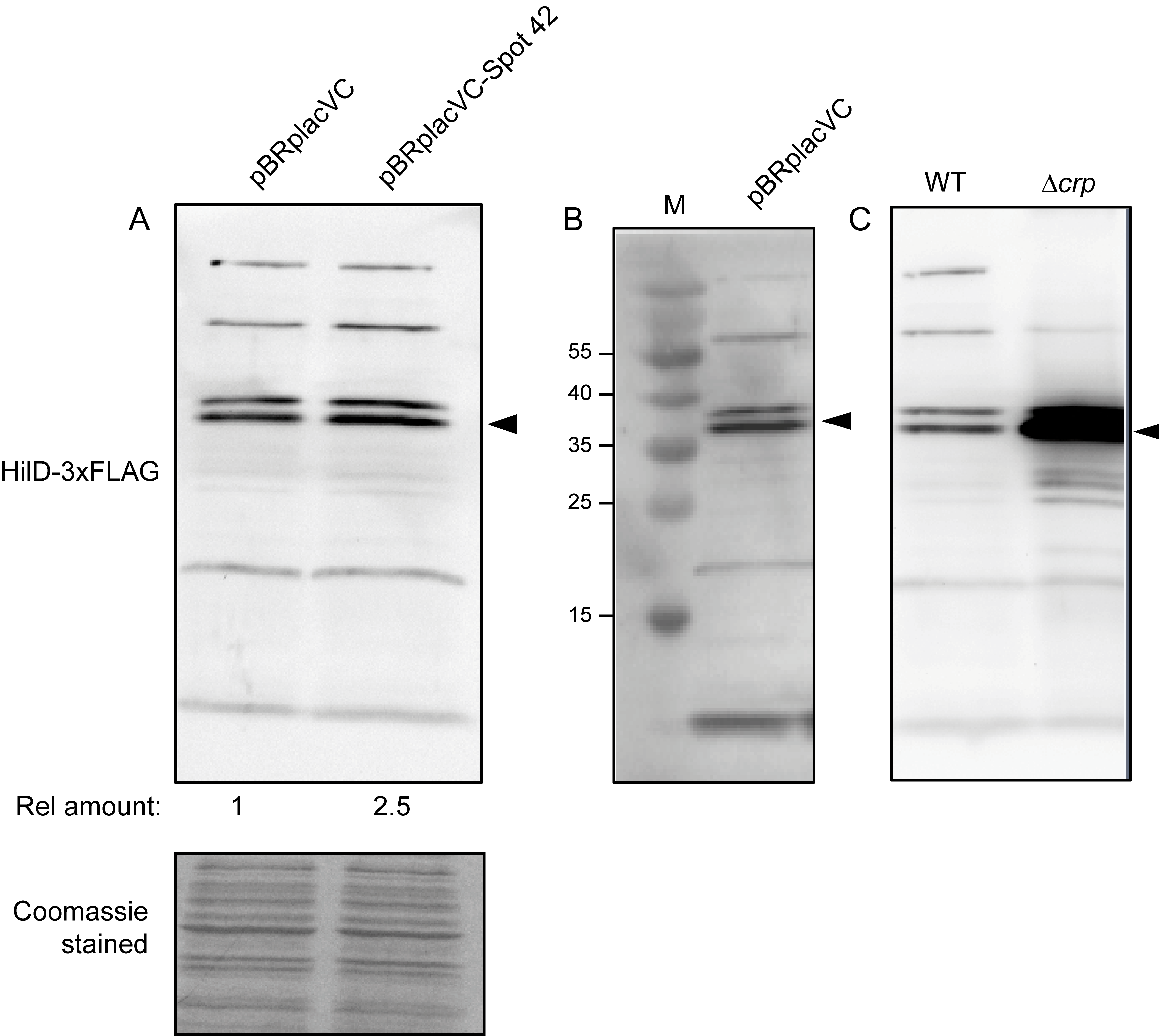

Supplement: S6 Fig — A. Immunodetection of HilD-3xFLAG was performed on whole cell extracts from cultures of the wild type (WT) strain (+UTR) carrying either the pBRplacVC (control vector, reference value) or pBRplac-Spot 42. Coomassie Blue staining of the whole cell extracts serve as loading controls. B. Merged image of white light caption for detection of the molecular mass marker and the chemiluminiscence detected bands in an extract from WT carrying the pBRplacVC. Molecular mass markers in kDa. C. The band corresponding to HilD-3xFLAG (indicated with an arrowhead) is easily identified as the protein band over-accumulated in Δcrp as compared to WT. (TIF) [file pgen.1007401.s006.tif]

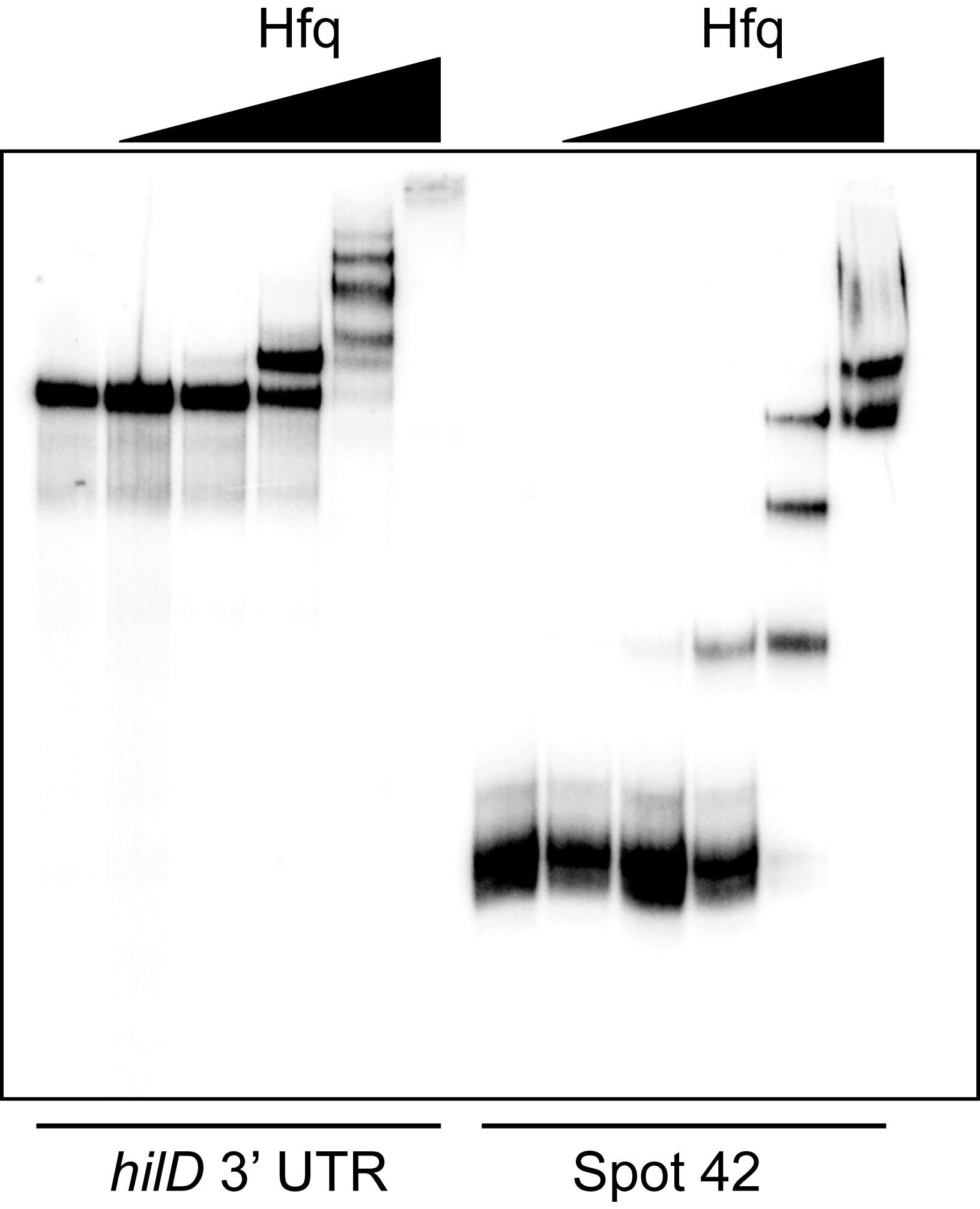

Supplement: S7 Fig — In vitro transcribed RNA was radiolabeled. 4 nM of the radiolabeled RNA was incubated with increasing concentration of purified Hfq (0, 1.3, 4, 13, 40, 130 nM) and subjected to electrophoresis in a native gel. Band shift was observed upon drying and exposure of the gel. (TIF) [file pgen.1007401.s007.tif]

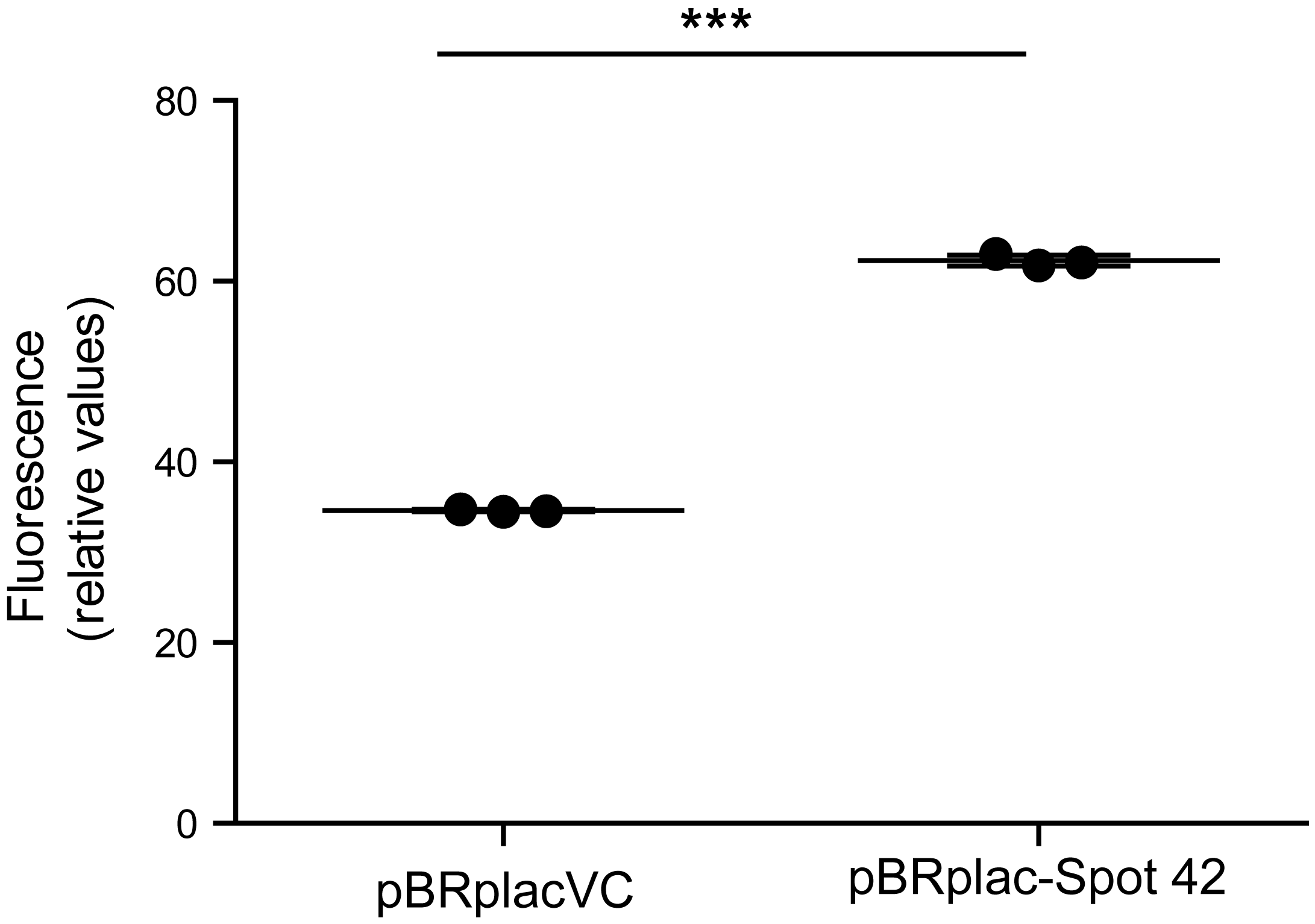

Supplement: S8 Fig — GFP fluorescence assessment by flow cytometry of GFP-hilD 3’UTR upon overexpression of the sRNA Spot 42. Cultures of Δspf strains carrying the construct pXG1gfp-hilD3’UTR in presence (pBRplac-Spot42) or absence of the sRNA Spot 42 (pBRplacVC) grown in LB at 37°C up to an OD600nm of 0.4. Data from three independent experiments are averaged and the standard deviation is shown. ***, p< 0.001. (TIF) [file pgen.1007401.s008.tif]

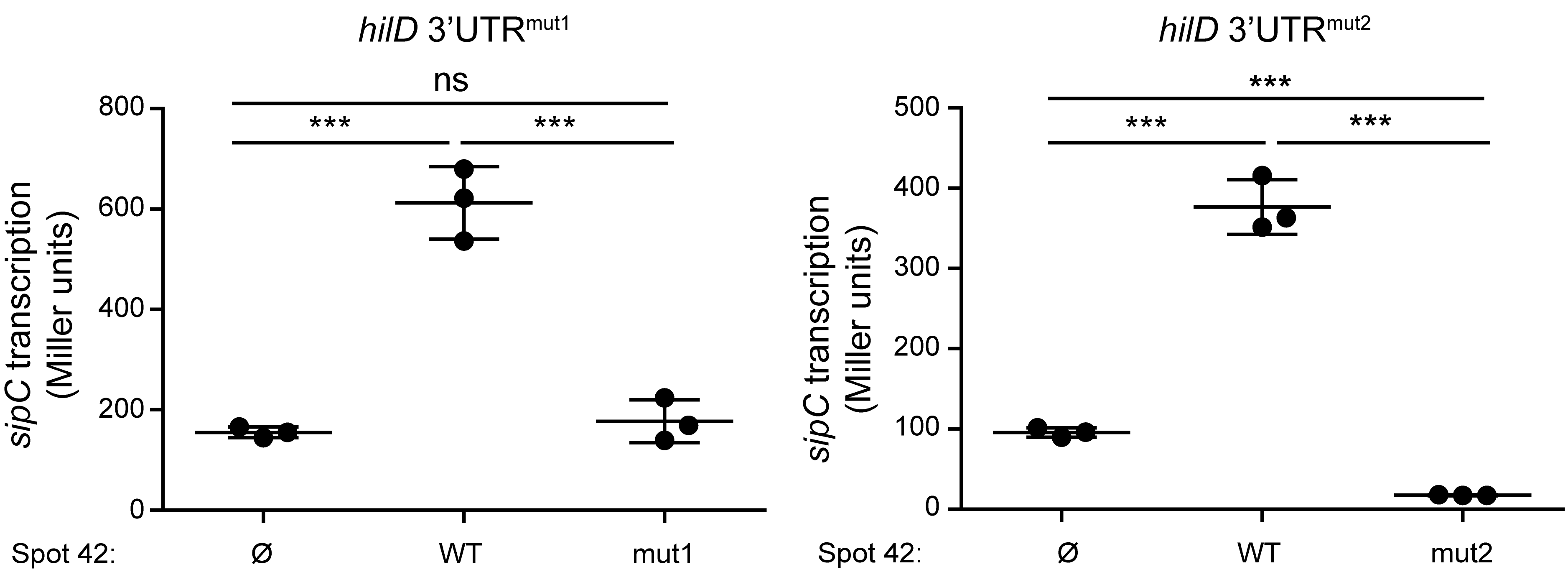

Supplement: S9 Fig — Transcriptional expression of sipC-lacZ was monitored in two different hilD backgrounds: hilD 3’UTRmut1 and hilD 3’UTRmut2. sipC-lacZ expression was assessed upon overexpression of either Spot 42WT, Spot 42mut1 and Spot 42mut2. β-galactosidase activity was determined for three independent cultures, average and standard deviation are shown. ***, p< 0.001; ns, not significant. In all cases, bacterial cultures were grown in LB at 37°C up to an OD600nm of 0.4. (TIF) [file pgen.1007401.s009.tif]

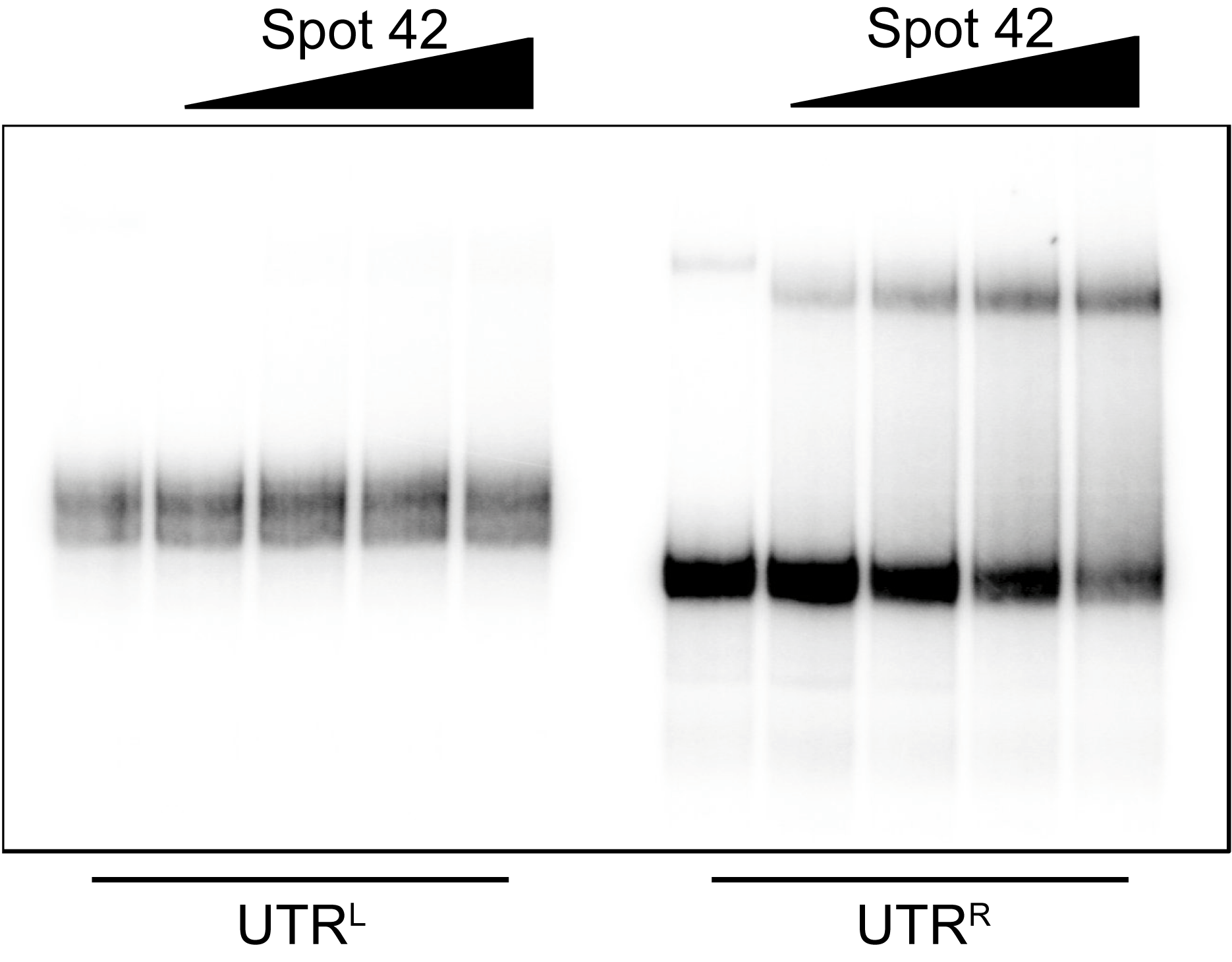

Supplement: S10 Fig — EMSA assay using 4 nM of either UTRL or UTRR fragments incubated with increasing concentration (0, 56, 280, 560, 1700 nM) of Spot 42 RNA. All RNA molecules used were obtained by T7 in vitro transcription. Samples were subjected to electrophoresis in a native gel and band shift was observed upon drying and exposure of the gel. (TIF) [file pgen.1007401.s010.tif]

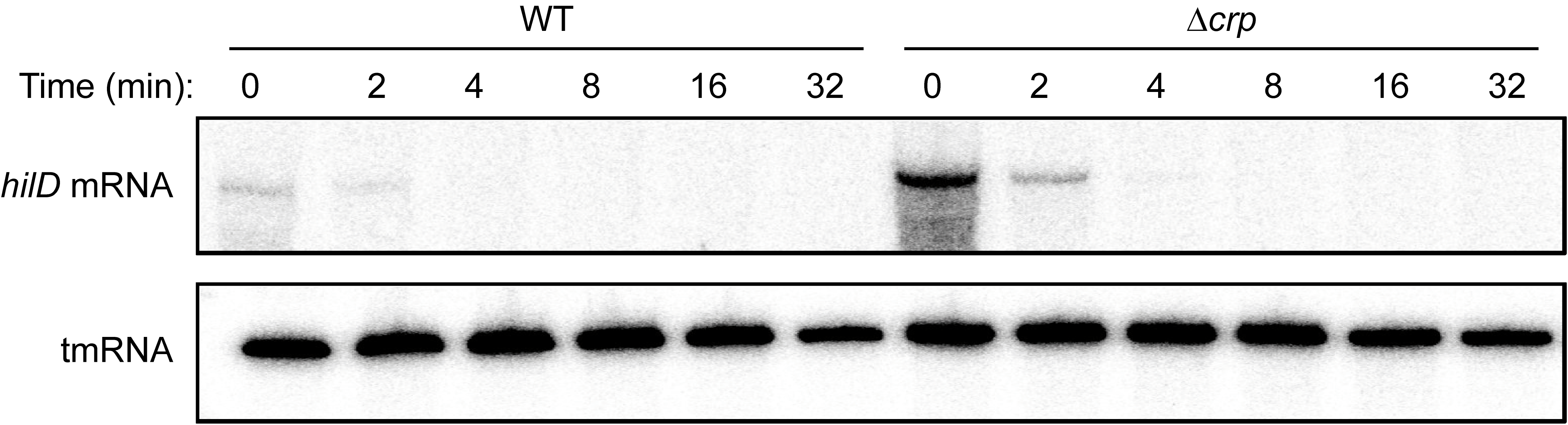

Supplement: S11 Fig — hilD mRNA was detected by Northern blot. Culture of wild type (WT) and Δcrp were grown to mid-logarithmic phase (OD600nm 0.4), rifampicin was added (500 μg/ml) and samples were taken for total RNA extraction at 2, 4, 8, 16 and 32 min. Samples before rifampicin addition (time 0) were taken. RNA radiolabeled probe complementary to the first 300 nt of hilD mRNA was generated by in vitro T7 RNA transcription and used for hilD mRNA detection. tmRNA was detected as loading control. Full length image of the Northern blot is shown in S12 Fig. (TIF) [file pgen.1007401.s011.tif]
