# Supplementary material for: CRP-cAMP mediates silencing of Salmonella virulence at the post-transcriptional level
Source: PLoS Genet. 2018 Jun 7;14(6):e1007401. doi: 10.1371/journal.pgen.1007401 (PMC5991649; doi:10.1371/journal.pgen.1007401)
Supplement: S12 Fig — (PDF) [file pgen.1007401.s012.pdf]

Figure S12. Compendium of uncropped images used to generate Figures 1-6 and Figure S11.

- **<u>Images Figure 1 (HilA-3xFLAG immunodetection)</u>**

A. Merged image of the white light caption for detection of the molecular mass marker and the chemiluminiscence detected bands during immunodetection of HilA-3xFLAG in WT whole cell extract (OD 2.0). B. Uncropped image of the Western blot shown in Fig. 1 panel B. C. Uncropped image of the Western blot shown in Fig. 1 panel C.). D. Uncropped image of the Western blot shown in Fig. 1 panel D. An arrowhead indicate the band corresponding to SopE.

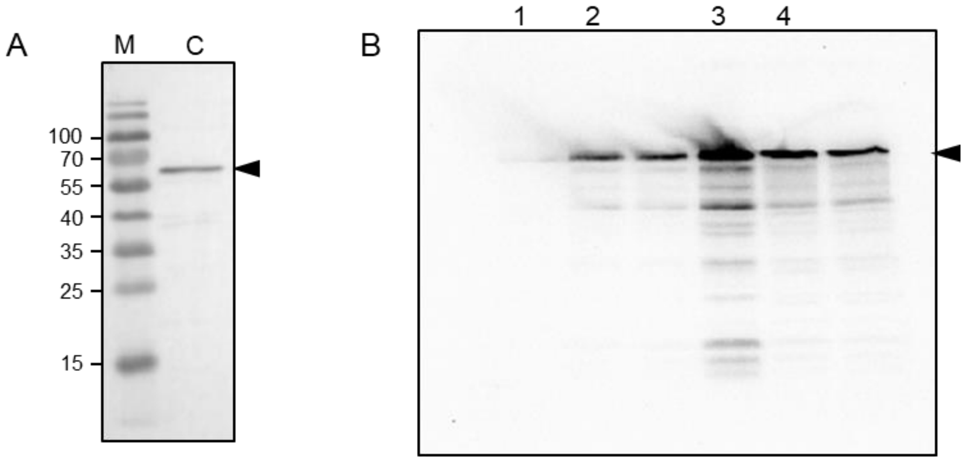

M: molecular mass marker (kDa)
C (control): whole cell extracts
of WT strain OD 2.0

1: WT whole cell extract, OD 0.4
2: Δ*crp* whole cellextract, OD 0.4
3: WT whole cell extract, OD 2.0
4: Δ*crp* whole cell extract, OD 2.0

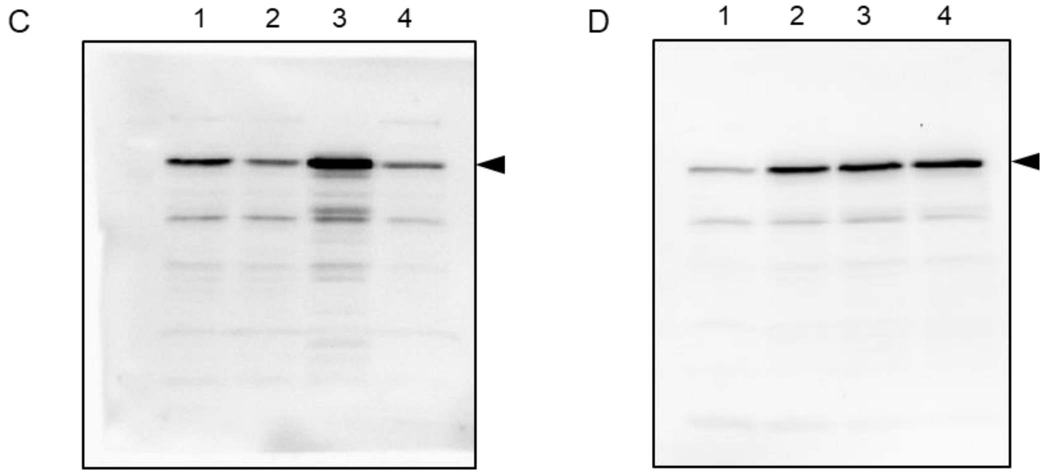

1: WT whole cell extract (- cAMP)
2: WT whole cell extract (+ cAMP)
3: Δ*crp* whole cellextract (- cAMP)
4: Δ*crp* whole cellextract (+ cAMP)

1: WT whole cell extract (- O/E CpdA)
2: WT whole cell extract (+ O/E CpdA)
3: Δ*crp* whole cellextract (- O/E CpdA)
4: Δ*crp* whole cellextract (+ O/E CpdA)

- **<u>Image Figure 2 A (SopE immunodetection)</u>**

Merged image of the white light caption for detection of the molecular mass marker and the chemiluminiscence detected bands (uncropped Western image shown in Fig. 2 panel A). An arrowhead indicate the band corresponding to SopE.

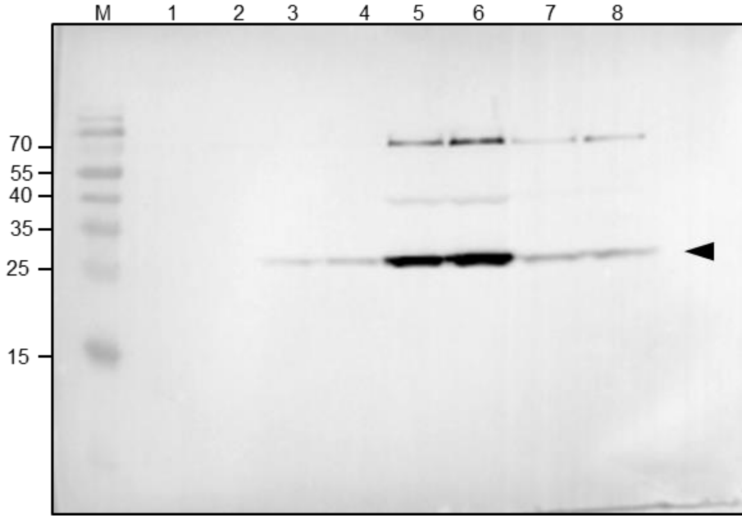

M: molecular mass marker (kDa)
1, 2: WT secreted protein extract, OD 0.4
3, 4: Δ*crp* secreted protein extract, OD 0.4
5, 6: WT secreted protein extract, OD 2.0
7, 8: Δ*crp* secreted protein extract, OD 2.0

- **Image Figure 2 B (SipA-3xFLAG immunodetection)**

A. Merged image of the white light caption for detection of the molecular mass marker and the chemiluminiscence detected bands during immunodetection of SipA-3xFLAG in WT whole cell extract (OD 2.0).

B. Uncropped image of the Western blot shown in Fig. 2 panel B.

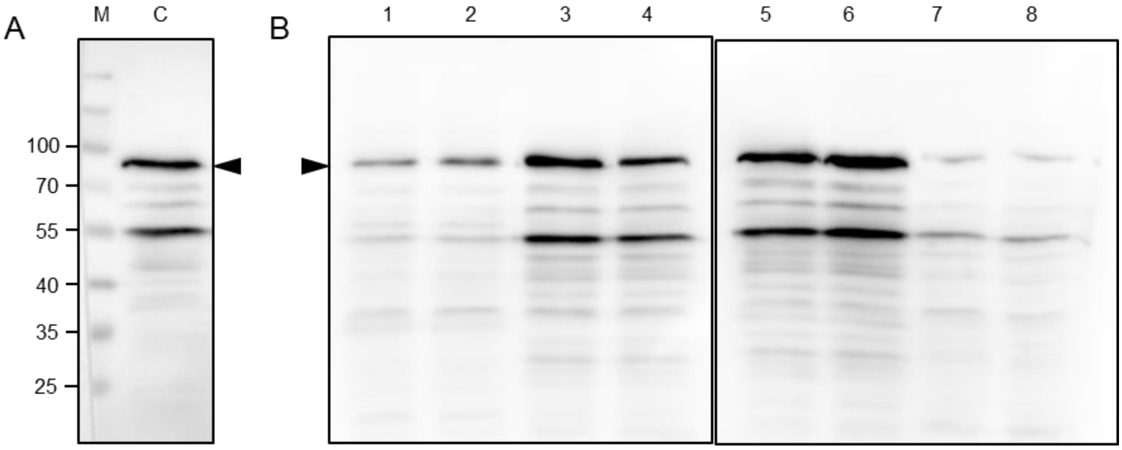

M: molecular mass marker (kDa)
1, 2: WT secreted protein extract, OD 0.4
3, 4: Δ*crp* secreted protein extract, OD 0.4
5, 6: WT secreted protein extract, OD 2.0
7, 8: Δ*crp* secreted protein extract, OD 2.0

- **Image Figure 3 D and Fig. S11**

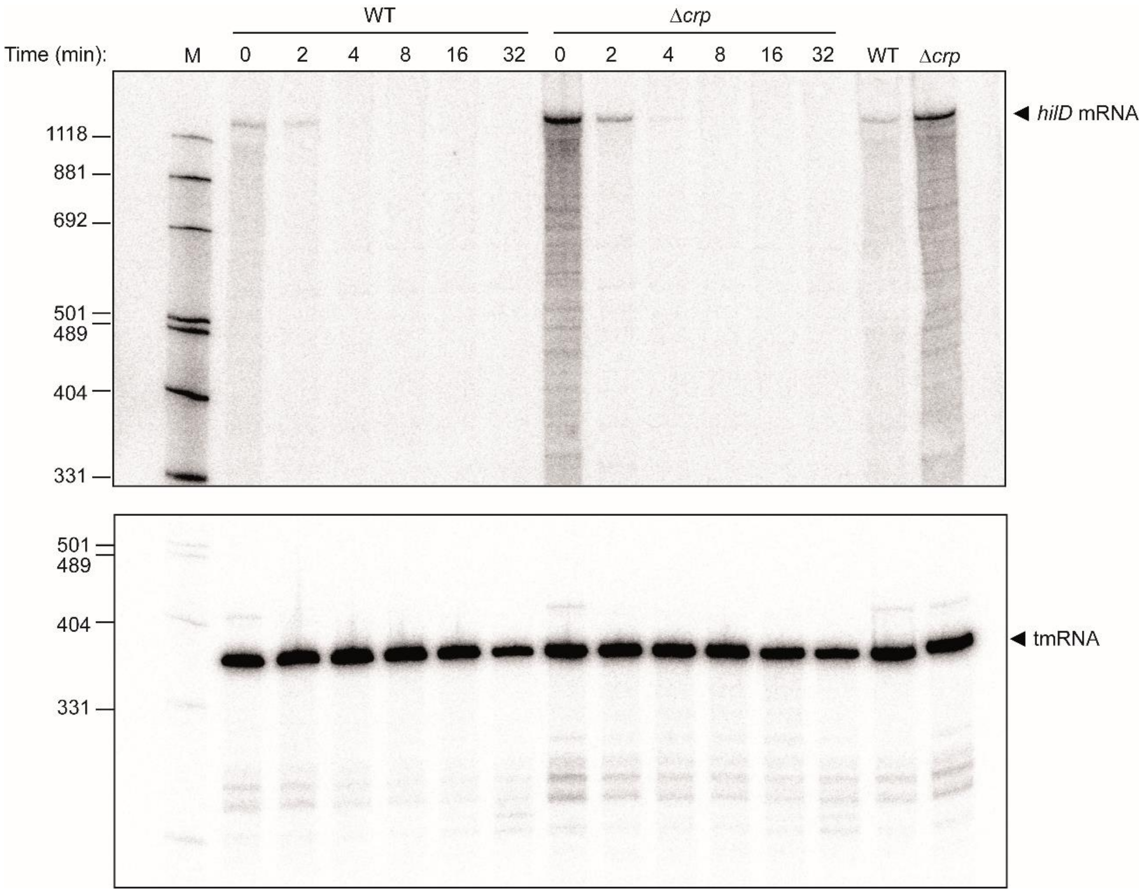

Northern blots to detect *hilD* mRNA (upper panel) and tmRNA (lower panel).

M: molecular mass marker indicated, sizes in nucleotides.

Lanes 2-13, used to generate Figure S11. Last two lanes used to generate panel Figure 3D.

- **Image Figure 4 B (HilD-3xFLAG immunodetection)**

A. Merged image of A. Merged image of the white light caption for detection of the molecular mass marker and the chemiluminiscence detected bands during immunodetection of HilD-3xFLAG in WT whole cell extract (-UTR).

B. Uncropped image of the Western blot shown in Fig. 4 panel B.

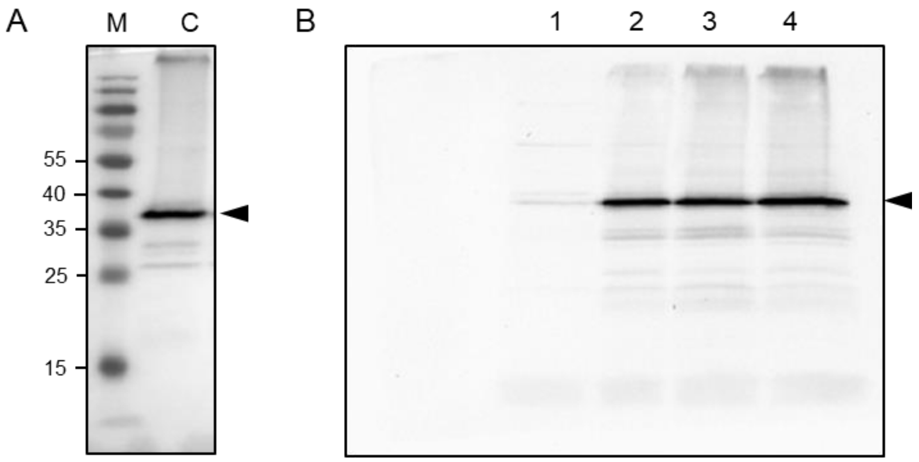

M: molecular mass marker (kDa)
C (control): Wt whole cell extract (-UTR)

1: Wt whole cell extract (+UTR)
2: Δ*crp* whole cell extract (+UTR)
3: Wt whole cell extract (-UTR)
4: Δ*crp* whole cell extract (-UTR)

- **Image Figure 4 C (SipA-3xFLAG immunodetection)**

Uncropped images of the Western blots shown in Fig. 4 panel C. The left image is a merged image of the white light caption for detection of the molecular mass marker and the chemiluminiscence detected bands during immunodetection of SipA-3xFLAG.

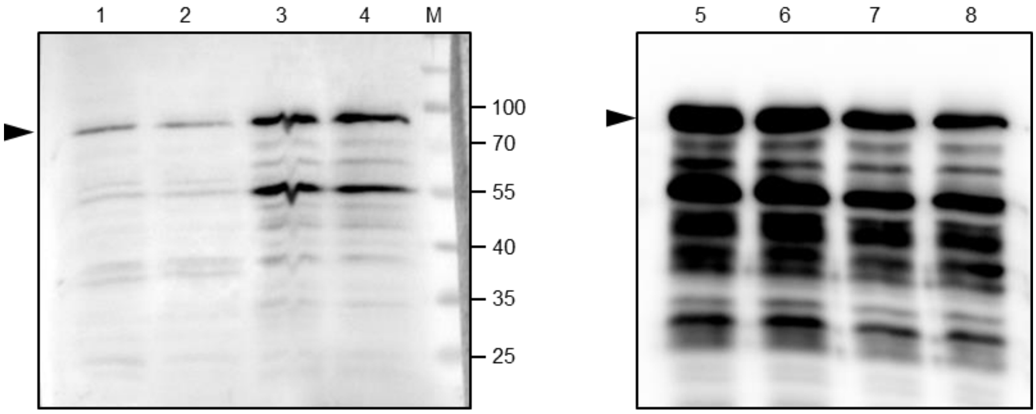

1, 2: Wt whole cell extract (+UTR)
3, 4: Δ*crp* whole cell extract (+UTR)
M: molecular mass marker (kDa)
5, 6: Wt whole cell extract (-UTR)
7, 8: Δ*crp* whole cell extract (-UTR)

- **Images Figure 5 C (Northern Spot42 detection)**

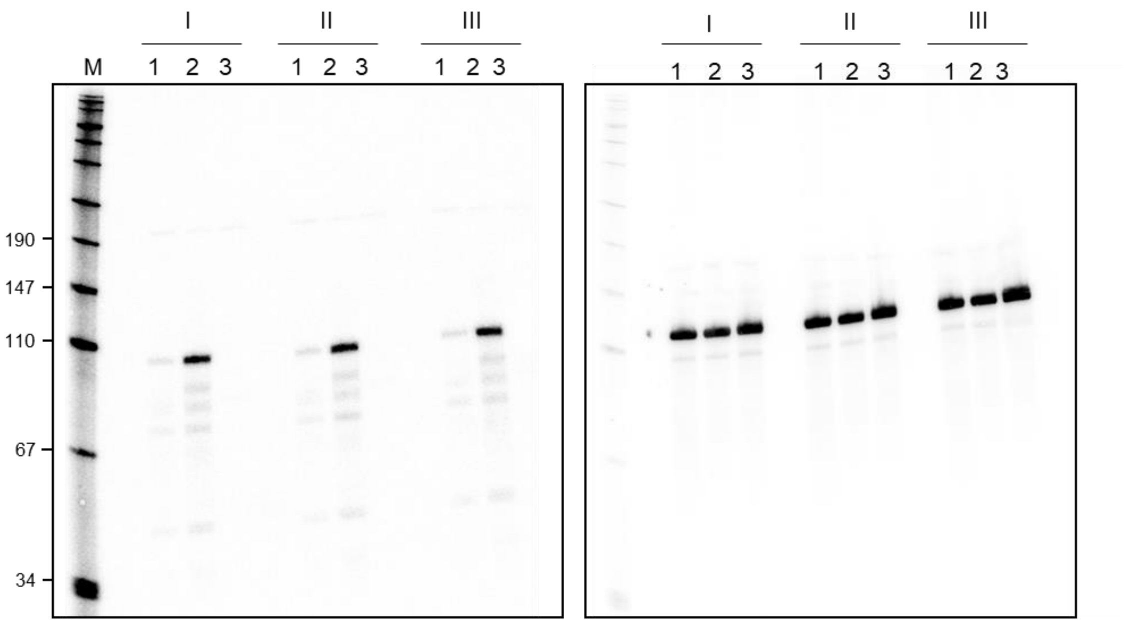

Northern blot analysis for Spot 42 sRNA (left panel) and 5S rRNA (right panel). Total RNA samples were extracted from cultures of the wild-type (1), Δ*crp* (2) and Δ*spf* (3) strain grown in LB at 37ºC up to an OD_{600nm} of 0.4. 5S rRNA served as loading control. Samples from three independent cultures were obtained (I, II and III). M: molecular size marker in (nt).

- **Images Figure 6 Panel D (Northern Spot 42 detection)**

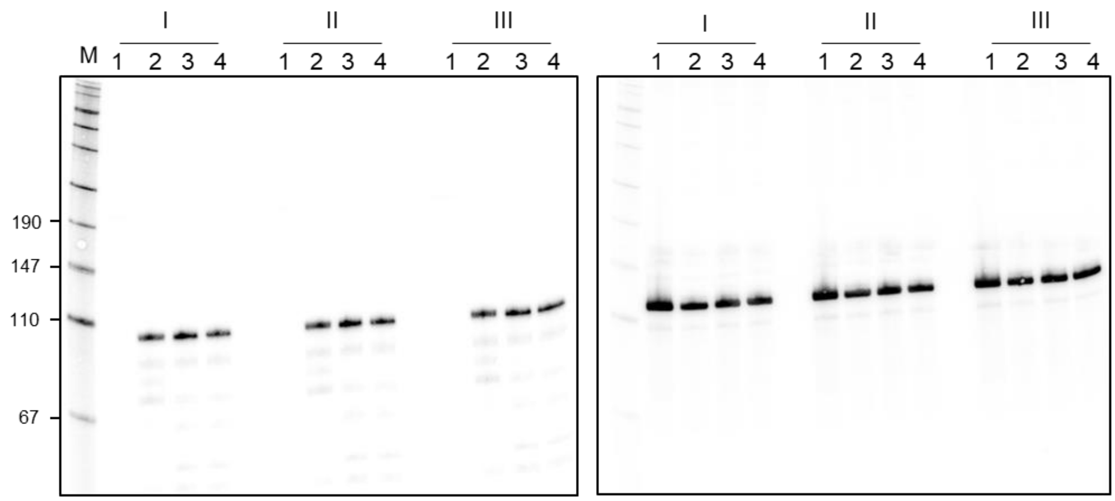

Northern blot analysis for Spot 42 sRNA (left panel) and 5S rRNA (right panel). Total RNA samples were extracted from cultures of the Δ*spf* strain carrying the control vector pBRplacVC (1) or derivatives to over-express Spot 42 (2), Spot42^{mut1} (3) and Spot 42^{mut2} (4). Strains were grown in LB at 37ºC up to an OD_{600nm} of 0.4. Samples from three independent cultures were obtained (I, II and III). M: molecular size marker in (nt).
