# Supplementary material for: CRP-cAMP mediates silencing of Salmonella virulence at the post-transcriptional level
Source: PLoS Genet. 2018 Jun 7;14(6):e1007401. doi: 10.1371/journal.pgen.1007401 (PMC5991649; doi:10.1371/journal.pgen.1007401)
Supplement: S1 Table — (PDF) [file pgen.1007401.s013.pdf]

Table S1. Strains and plasmids used in this work

Strains	Genotype	Source
SV5015	*Salmonella enterica* serovar Typhimurium SL1344 His+	[1]
TGC200	SV5015 Δ*crp*:frt	This study
TGC201	SV5015 Δ*cya*:frt	This study
SV5015UB2	SV5015 *hilA-lacZ*	[2]
TGC202	SV5015 Δ*crp*:frt *hilA-lacZ*	This study
JPTM7	SV5015 HilA-3xFLAG::*km*	[3]
TGC203	SV5015 Δ*crp*:frt HilA-3xFLAG::*km*	This study
TGC244	SV5015 Δ*cya*:frt HilA-3xFLAG::*km*	This study
TGC204	SV5015 HilA-3xFLAG*::km* pTRC99a VC	This study
TGC205	SV5015 Δ*cya*:Frt HilA-3xFLAG*::km* pTRc99aVC	This study
TGC206	SV5015 HilA-3XFLAG*::km* pTRc99a CpdA	This study
TGC207	SV5015 Δ*cya*:*frt* HilA-3XFLAG*::km* pTRc99a CpdA	This study
MHS2	SV5015 SipA-3XFLAG	[4]
TGC208	SV5015 Δ*crp*:*frt* SipA-3xFLAG	This study
TGC14	SV5015 Δ*rtsa*::*cm hilA-lacZ*	This study
TGC209	SV5015 Δ*crp*:frt Δ*rtsA*::*cm hilA-lacZ*	This study
TGC10	SV5015 Δ*hilD*::*cm hilA-lacZ*	This study
TGC210	SV5015 Δ*crp*:*frt* Δ*hilD*::*cm hilA-lacZ*	This study
TGC8	SV5015 Δ*hilC*:*frt hilA-lacZ*	This study
TGC211	SV5015 Δ*crp*:frt Δ*hilC*:frt *hilA-lacZ*	This study
TGC212	SV5015 *hilC-lacZ*	This study
TGC213	SV5015 Δ*crp*:*frt hilC-lacZ*	This study
TGC214	SV5015 Δ*crp*:*frt* Δ*hilD*::*cm hilC-lacZ*	This study
TGC35	SV5015 *hilD*1235-*lacZ*	This study
TGC215	SV5015 Δ*crp*:*frt hilD*1235-*lacZ*	This study
TGC216	SV5015 Δ*crp*:*frt* Δ*hilC*:*frt hilD*1235-*lacZ*	This study
TH20428	*Salmonella enterica* serovar Typhimurium LT2 HilD-3xFLAG	[5]
TGC217	SV5015 HilD-3xFLAG::*km*	This study
TGC218	SV5015 Δ*crp*:*frt* HilD-3xFLAG*::km*	This study
TGC219	SV5015 HilD-3xFLAG:*frt*	This study
TGC220	SV5015 Δ*crp*:*frt* HilD-3xFLAG:*frt*	This study
SV6190	SV5015 *hilD*ΔUTR:*km*	[6]
TGC221	SV5015 *hilD*ΔUTR:*frt* SipA-3xFLAG	This study
TGC222	SV5015 Δ*crp*:*frt hilD*ΔUTR:*frt* SipA-3xFLAG	This study
TGC25	SV5015 *hilD*76-*lacZ*	This study
TGC223	SV5015 Δ*crp*:*frt hilD*76-*lacZ*	This study
TGC38	SV5015 *hilD*965-*lacZ*	This study
TGC224	SV5015 Δ*crp*:*frt hilD*965-*lacZ*	This study
SV5293	14028 Φ(*sipC'-lacZ'*)	[7]
TGC40	SV5015 *Φ(sipC'-lacZ')*	This study

TGC225	SV5015 Δ*crp*:*frt* Φ(sipC'-lacZ')	This study
TGC226	SV5015 *hilD*ΔUTR:*frt Φ(sipC'-lacZ')*	This study
TGC227	SV5015 Δ*crp*:*frt hilD*ΔUTR:*frt Φ(sipC'-lacZ')*	This study
TGC228	SV5015 Δ*hfq*::*cm hilD*1235-*lacZ*	This study
TGC229	SV5015 Δ*crp*:*frt* Δ*hfq*::*cm hilD*1235-*lacZ*	This study
TGC230	SV5015 Δ*hfq*::*cm Φ(sipC'-lacZ')*	This study
TGC231	SV5015 Δ*crp*:*frt* Δ*hfq*::*cm* Φ(sipC'-lacZ')	This study
TGC232	SV5015 *hilD*965-*lacZ* pBRplacVC	This study
TGC233	SV5015 *hilD*965-*lacZ* pBRplac Spot 42	This study
TGC234	SV5015 *hilD*1235-*lacZ* pBRplacVC	This study
TGC235	SV5015 *hilD*1235-*lacZ* pBRplac Spot 42	This study
TGC236	SV5015 Δ*hfq*::*cm hilD*1235-*lacZ* pBRplacVC	This study
TGC237	SV5015 Δ*hfq*::*cm hilD*1235-*lacZ* pBRplac Spot 42	This study
SV5961	SV5015 *rne*537::*cm*	[7]
TGC254	SV5015 *rne*537::*cm hilD*1235-*lacZ* pBRplacVC	This study
TGC255	SV5015 *rne*537::*cm hilD*1235-*lacZ* pBRplac Spot 42	This study
TGC238	SV5015 *Φ(sipC'-lacZ')* pBRplacVC	This study
TGC239	SV5015 *hilD*ΔUTR:*frt Φ(sipC'-lacZ')* pBRplacVC	This study
TGC240	SV5015 *Φ(sipC'-lacZ')* pBRplac Spot 42	This study
TGC241	SV5015 *hilD*ΔUTR:*frt Φ(sipC'-lacZ')* pBRplac Spot 42	This study
TGC242	SV5015 Δ*spf*::*cm hilD*1235-*lacZ*	This study
TGC243	SV5015 Δ*crp*:*frt* Δ*spf*::*cm hilD*1235-*lacZ*	This study
TGC246	SV5015 *Φ(sipC'-lacZ')* pBRplac Spot 42^{Mut1}	This study
TGC247	SV5015 *Φ(sipC'-lacZ')* pBRplac Spot 42^{Mut2}	This study
TGC248	SV5015 *hilD*UTR^{Mut1} *Φ(sipC'-lacZ')* pBRplacVC	This study
TGC249	SV5015 *hilD*UTR^{Mut1} *Φ(sipC'-lacZ')* pBRplac Spot 42	This study
TGC250	SV5015 *hilD*UTR^{Mut1} *Φ(sipC'-lacZ')* pBRplac Spot 42^{Mut1}	This study
TGC251	SV5015 *hilD*UTR^{Mut2} *Φ(sipC'-lacZ')* pBRplacVC	This study
TGC252	SV5015 *hilD*UTR^{Mut2} *Φ(sipC'-lacZ')* pBRplac Spot 42	This study
TGC253	SV5015 *hilD*UTR^{Mut2} *Φ(sipC'-lacZ')* pBRplac Spot 42^{Mut2}	This study

**Plasmids**	**Antibiotic resistance**	**Source**
pTRc99a	Amp^R	[8]
pTRc99a CpdA	Amp^R	This study
pXG1	Cm^R	[9]
pXG1 *gfp*-3'UTR	Cm^R	This study
pBRplac	Amp^R	[10]
pBRplac Spot 42	Amp^R	This study
pBRplac Spot 42^{Mut1}	Amp^R	This study
pBRplac Spot 42^{Mut2}	Amp^R	This study

References

1. Vivero A, Baños RC, Mariscotti JF, Oliveros JC, García-del Portillo F, Juárez A, et al. Modulation of horizontally acquired genes by the Hha-YdgT proteins in *Salmonella enterica* serovar Typhimurium. J Bacteriol. 2008;190: 1152–1156. doi:10.1128/JB.01206-07
2. Baños RC, Vivero A, Aznar S, García J, Pons M, Madrid C, et al. Differential regulation of horizontally acquired and core genome genes by the bacterial modulator H-NS. PLoS Genet. 2009;5: e1000513. doi:10.1371/journal.pgen.1000513
3. Bustamante VH, Martínez LC, Santana FJ, Knodler LA, Steele-Mortimer O, Puente JL. HilD-mediated transcriptional cross-talk between SPI-1 and SPI-2. Proc Natl Acad Sci U S A. 2008;105: 14591–14596. doi:10.1073/pnas.0801205105
4. Hüttener M, Dietrich M, Paytubi S, Juárez A. HilA-like regulators in *Escherichia coli* pathotypes: the YgeH protein from the enteroaggregative strain 042. BMC Microbiol. 2014;14: 268. doi:10.1186/s12866-014-0268-5
5. Mouslim C, Hughes KT. The effect of cell growth phase on the regulatory cross-talk between flagellar and Spi1 virulence gene expression. PLoS Pathog. 2014;10: e1003987. doi:10.1371/journal.ppat.1003987
6. López-Garrido J, Puerta-Fernández E, Casadesús J. A eukaryotic-like 3' untranslated region in *Salmonella enterica hilD* mRNA. Nucleic Acids Res. 2014;42: 5894–5906. doi:10.1093/nar/gku222
7. López-Garrido J, Casadesús J. Regulation of *Salmonella enterica* pathogenicity island 1 by DNA adenine methylation. Genetics. 2010;184: 637–649. doi:10.1534/genetics.109.108985
8. Amann E, Ochs B, Abel KJ. Tightly regulated tac promoter vectors useful for the expression of unfused and fused proteins in *Escherichia coli*. Gene. 1988;69: 301–315.
9. Urban JH, Vogel J. Translational control and target recognition by *Escherichia coli* small RNAs in vivo. Nucleic Acids Res. 2007;35: 1018–1037. doi:10.1093/nar/gkl1040
10. Guillier M, Gottesman S. Remodelling of the *Escherichia coli* outer membrane by two small regulatory RNAs. Mol Microbiol. 2006;59: 231–247. doi:10.1111/j.1365-2958.2005.04929.x
