# Supplementary material for: CRP-cAMP mediates silencing of Salmonella virulence at the post-transcriptional level
Source: PLoS Genet. 2018 Jun 7;14(6):e1007401. doi: 10.1371/journal.pgen.1007401 (PMC5991649; doi:10.1371/journal.pgen.1007401)
Supplement: S2 Table — (PDF) [file pgen.1007401.s014.pdf]

S2 Table: Oligonucleotides used in this work

Primers	Sequence
*crp*_P1	ATGGTGCTTGGCAAACCGCAAACAGACCCGACTCTTGAATGGGTGTAGGCTGGAGCTGCTTC
*crp*_P2	TTAGCGTGTCTTAATAACCAGACGATTACTCTGTTTTACCTCCATATGAATATCCTCCTTAGT
*cya*_P1	TTGTACCTCTATATTGAGACTCTGAAACAGAGACTGGATGCCGTGTAGGCTGGAGCTGCTTC
*cya*_P2	TTACGAAAAATACTGCTGCAATAGCGGCGCGTCATGATCCTGCATATGAATATCCTCCTTAGT
*cpdA_XbaI*_Fw	CGCTCTAGAGTTTATTAGCGTCGTGAAACC
*cpdA_SalI*_Rev	GCGGTCGACATCAGTGATGGTGATGGTGATGGTATCCTTCCGAAGCGGTATC
*hilD*_qPCR_Fw	GCCAGAAGAG AGGTATTTG
*hilD*_qPCR_Rev	CAGTAAGCAGGAACAGCAG
*hilC*_qPCR_Fw	CTCACCCGCAAATGGTCAC
*hilC*_qPCR_Rev	GCCTGATTCATACGAGCATC
*rtsA*_qPCR_Fw	GTATATTACGGCATCAGGGC
*rtsA*_qPCR_Rev	GCCTGTTTCTATTGGCGC
*gapA*_qPCR_Fw	GTCCGTCTAAAGACAACACC
*gapA*_qPCR_Rev	CATCAGACCTTCGATGATGC
*hilC*_P1	ATGGTATTGCCTTCAATGAATAAATCAGTTGAGGCCATTAGCGTGTAGGCTGGAGCTGCTTC
*hilC*_P2	TCAATGGTTCATTGTACGCATAAAGCTAAGCGGTGTAATCTTCATATGAATATCCTCCTTAGT
*rtsA*_P1	GCACATTTAATAAAAGGAAATTATCATGCTAAAAGTATTTAATCCCTCACCGTGTAGGCTGGAGCTGCTTC
*rtsA*_P2	TCTTATACTGCATTGTCAGATATCTCAATTAACATATTGATGACGAGAGGCATATGAATATCCTCCTTAGT
*hilD*_P1+76	ATGGAAAATGTAACCTTTGTAAGTAATAGTCATCAGCGTCCTGTGTAGGCTGGAGCTGCTTC
*hilD*_P2+76	TTAATGGTTCGCCATTTTTATGAATGTCGATGGCGTAGTTTTCATATGAATATCCTCCTTAGT
*hilD*_P1+1235	GATATTGCCTTATTCACATCGTAAGAATTCGTCCAGATGACACTATCTCCGTGTAGGCTGGAGCTGCTTC
*hilD*_P2+1235	TATAAATATGAATAAAATGCCGGCCTTAATCCACAGGGTTAAAGCCGGAACATATGAATATCCTCCTTAGT
*hfq*_P1	GTACAATTGAGACGTATCGTGCGCAATTTTTCAGAATCGAGTGTAGGCTGGAGCTGCTTC
*hfq*_P2	CCCGACATGGATAAACAGCGCGTGAACTTATTCAGTCTCTTGATATGAATATCCTCCTTA
*gfp_Nhe*I_Fw	GCGGCTAGCAAAGGAGAAGAACTTTTCACTGG
*gfp_hilD*_rev	CAGATACAAAAAATGTTATTTGTAGAGCTCATCC
*hilD_gfp*_Fw	GAGCTCTACAAATAACATTTTTTGTATCTGTCACTTAAG
*hilD*_xbaI_rev	GCGTCTAGATGCCTGGCAGAAAGCTAACAAGC
*spf*_P1	GTAATTAAGCAACATAAGCACGGGGGTTTTGTGATGGGTATTGTGTAGG
*spf*_P2	GTCTTATCCGGCCTACGGTGTGAGCGAAACTTTTCTTGCGCACATATGAATATCCTCCTTA
*spf_AatII*_Fw	CGCGACGTCGTAGGGTACAGAGGTAAGATGTTC
*spf_EcoRI*_Rev	CGCGAATTCGTCTTATCCGGCCTACGGTG

*spf*_probe	CAAATCCGATTACGTGAAGT
*spf*_mut1_Fw	GACCTTTTACTTCACCATATCGGATTTGGCTGAATATTTTAG
*spf*_mut1_rev	CTAAAATATTCAGCCAAATCCGATATGGTGAAGTAAAAGGTC
*spf*_mut2_Fw	GACCTTTTACTTCACGTAATCCACTTTGGCTGAATATTTTAG
*spf*_mut2_rev	CTAAAATATTCAGCCAAAGTGGATTACGTGAAGTAAAAGGTC
*hilD*UTR_*xbaI*_Fw	CGCTCTAGAGATACAAGCAAAGGTTGCAGTAAC
*hilD*UTR_*sacI*_rev	CGCGAGCTCCTCGAAGATTTCCACTAAATGACC
*hilD*UTR_mut1_Fw	GGTAATTTAAAGTAAGGCTGATATGATAACACGATTTTTGTG
*hilD*UTR_mut1_rev	CACAAAAATCGTGTTATCATATCAGCCTTACTTTAAATTACC
*hilD*UTR_mut2_Fw	GGTAATTTAAAGTAAGGTGGATTATATAACACGATTTTTGTG
*hilD*UTR_mut2_rev	CACAAAAATCGTGTTATATAATCCACCTTACTTTAAATTACC
*hilD*UTR_T7_fw	TAATACGACTCACTATAGGGCTACGCCATCGACATTCATAA
*hilD*UTR^{R}_T7_Fw	TAATACGACTCACTATAGGGACAGCTTATACTGATATCTATGG
*hilD*UTR^{L}_T7_rev	CCATAGATATCAGTATAAGCTGTC
*hilD*UTR_T7_rev	GAATAAAATGCCGGCCTTAATC
*Spf*_T7_Fw	TAATACGACTCACTATAGGGTAGGGTACAGAGGTAAGATG
*Spf*_T7_rev	GTCTTATCCGGCCTACGGTG
*hilD*_riboprobeFw	ATGGAAAATGTAACCTTTGTAAG
hilD_riboprobeRev	GTTTTTTTAATACGACTCACTATAGGGAGGTATATCGAAATCCATGTGGC
tmRNA probe	TGGTGGAGCTGGCGGGAGTT
